# Inhibitory Effect of Dietary Defatted Rice Bran in an AOM/DSS-Induced Colitis-Associated Colorectal Cancer Experimental Animal Model

**DOI:** 10.3390/foods11213488

**Published:** 2022-11-02

**Authors:** Laleewan Tajasuwan, Aikkarach Kettawan, Thanaporn Rungruang, Kansuda Wunjuntuk, Pinidphon Prombutara, Chawanphat Muangnoi, Aurawan Kringkasemsee Kettawan

**Affiliations:** 1 Graduate Student in Doctor of Philosophy Program in Nutrition, Faculty of Medicine Ramathibodi Hospital and Institute of Nutrition, Mahidol University, Bangkok 10400, Thailand; 2 Institute of Nutrition, Mahidol University, Nakhon Pathom 73170, Thailand; 3 Department of Anatomy, Faculty of Medicine Siriraj Hospital, Mahidol University, Bangkok 10700, Thailand; 4 Department of Home Economics, Faculty of Agriculture, Kasetsart University, Bangkok 10900, Thailand; 5 OMICS Sciences and Bioinformatics Center, Faculty of Science, Chulalongkorn University, Bangkok 10330, Thailand

**Keywords:** defatted rice bran, colitis-associated colorectal cancer, chemoprevention, anti-inflammation, anti-cell proliferation, health, health product

## Abstract

Defatted rice bran (DRB) is gaining immense popularity worldwide because of its nutritional and functional aspects. Emerging evidence suggests that DRB is a potential source of dietary fiber and phenolic compounds with numerous purported health benefits. However, less is known about its chemoprotective efficacy. In the present study, we determined and examined the nutrient composition of DRB and its chemopreventive effect on azoxymethane and dextran sulphate sodium (AOM/DSS)-induced colitis-associated colorectal cancer (CRC) in rats. The results showed the presence of several bioactive compounds, such as dietary fiber, phytic acid, and phenolic acids, in DRB. In addition, DRB supplementation reduced the progression of CRC symptoms, such as colonic shortening, disease activity index (DAI), and histopathological changes. Interestingly, a significant decrease was observed in total numbers of aberrant crypt foci (ACFs) and tumors with DRB supplementation. Furthermore, DRB supplementation suppressed the expression of pro-inflammatory cytokines (IL-6) and inflammatory mediators (NF-κB and COX-2) through the inactivation of the NF-κB signaling pathway. The administration of DRB revealed a negative effect on cancer cell proliferation by repressing the expression of nuclear β-catenin, cyclin D1, and c-Myc. These findings suggest that DRB supplementation mitigates chronic inflammation and cancer cell proliferation and delays tumorigenesis in rat AOM/DSS-induced colitis-associated CRC. Therefore, the establishment of DRB as a natural dietary food-derived chemopreventive agent has the potential to have a significant impact on cancer prevention in the global population.

## 1. Introduction

In recent years, colorectal cancer (CRC) has been the third most commonly diagnosed cancer and the second leading cause of cancer death globally. Based on projections for population growth, aging, and human development, the global number of new CRC cases is predicted to reach 3.2 million in 2040 [1,2]. CRC manifests as a process of genetic and morphological alterations in the colonic epithelium, beginning with the formation of aberrant crypt foci (ACFs) progressing to adenomas and finally to adenocarcinomas [3]. There is no specific cause of the development of human CRC. However, the etiology of the disease could depend on age, genomic instability, and hereditary and environmental factors, including gut microbial dysbiosis, chronic gut inflammation, and dietary factors (e.g., diets high in fat, added sugar, and protein) [4,5]. In particular, chronic gut inflammation and dietary factors have been cited as significant contributors to CRC development. Excessive production of inflammatory responses by environmental triggers leads to the infiltration of inflammatory cells.

The produced pro-inflammatory cytokines, such as interleukin-1, 6 (IL-1 and IL-6), and tumor necrosis factor-alpha (TNF-α), from inflammatory cells lead to intestinal epithelial cell damage, tight junction dysregulation, and loss of the intestinal barrier [6]. Subsequently, these cytokines activate the nuclear factor kappa-light-chain-enhancer of activated B cells (NF-κB) and cyclooxygenase-2 (COX-2), which are involved in inflammatory and carcinogenetic processes [7,8]. Moreover, the positive regulation of Wnt/β-catenin by the NF-κB pathway contributes to cancer development [9]. The Wnt/β-catenin signaling pathway is imperative for maintaining cell homeostasis in tissues by regulating cell proliferation, migration, differentiation, survival, and adhesion [10]. Consequently, the synergistic cooperation between NF-κB and the β-catenin transcription factor results in the accumulation of β-catenin in the cytosol, which is then translocated to the nucleus [9]. The expression of β-catenin in the nucleus has been linked to CRC, as it is a co-activator of T cell factor/lymphoid enhancer factor (TCF/LEF)-mediated transcription factor, which regulates the expression of Wnt target genes, such as cellular myelocytomatosis (c-Myc) and cyclin D1. It further regulates the cancer cell proliferation process [11,12]. All these conditions accelerate the growth and proliferation of cancer cells, leading to CRC, often beginning as abnormal growth, hyperplasia, and dysplasia of epithelial cells called ACFs. ACFs, preneoplastic lesions, are currently surrogate biomarkers for colon carcinogenesis and chemopreventive response to nutritional intervention [13]. Furthermore, large dysplastic ACFs can develop into adenomas and adenocarcinomas. Therefore, regulation of the inflammatory response is critical for slowing the process of CRC.

Numerous studies have reported that natural food components found in fruits, vegetables, legumes, and wholegrain products can prevent the development of CRC through antioxidant, anti-inflammatory, and anti-proliferative effects. Rice (*Oryza sativa* L.) is one of the most important crops in the developing world. After the rice milling process, rice bran is produced as a by-product, which consists of the outer layer of the rice grain, composed of a sub-aleurone layer, an aleurone layer, the seed coat, and the pericarp, and which has a yield of about 5–10%. Rice bran has been extensively used for rice bran oil production. In industrial rice bran oil production, large amounts of defatted rice bran (DRB), a by-product of the oil extraction process, is produced. DRB has a sweet taste, nutty aroma, and light-brown color [14,15]. Although some bioactive compounds (e.g., γ-oryzanol, tocopherols, tocotrienols, and phytosterols) are excluded during the oil extraction process, DRB still contains protein, starch, vitamins, minerals, dietary fiber, phytic acid, and some phenolic compounds.

Previous studies have suggested that essential nutrients present in DRB have the potential to inhibit CRC [16,17,18]. Dietary fiber plays a crucial role in preventing CRC. Its higher intake may impact the composition of gut microbiota in healthy adults. The members of gut microbiota produce short-chain fatty acids from dietary fiber, which affect the progression of CRC [19]. Phytic acid, a six-fold dihydrogen phosphate ester of inositol, also called inositol hexakisphosphate (IP6) or inositol polyphosphate, has been shown to inhibit ACF numbers and tumor formation in the distal colon [20] by suppressing the expression of β-catenin and pro-inflammatory cytokines in an animal model [21,22]. In addition, some phenolic compounds, including ferulic acid and *p*-coumaric acid, have shown antioxidant and anti-inflammatory effects associated with inhibition of the development of CRC [23,24]. According to the previous studies, these components help to delay the progression of CRC through their anti-inflammatory and anti-cancer cell proliferation activities. Hence, the components in DRB might be possible active ingredients in the treatment of colitis-associated CRC.

The health benefits of DRB are currently gaining more attention with its use as a commercial health product. However, research on the health benefits and use of DRB as a chemopreventive agent is still in its infancy. Therefore, in this study, we aimed to determine the nutritional composition of DRB and the chemopreventive effects of the nutrients it contains on an AOM/DSS-induced colitis-associated CRC model to produce evidence for further studies and applications.

## 2. Materials and Methods

### 2.1. Defatted Rice Bran (DRB)

DRB was procured from Thai Ruam Jai Vegetable Oil Co., Ltd. (Phra Nakhon Si Ayutthaya, Thailand). It was obtained from various Thai rice varieties from Suphan Buri, Phra Nakhon Si Ayutthaya, Chachoengsao, and Phitsanulok Provinces. After rice bran oil extraction, the DRB pellet was dried, milled, and separated into DRB flour with a mesh of 60 mm, and finally stored at −20 °C until further analysis.

### 2.2. Nutrient Analysis

All determinations were performed in triplicate and expressed as percentages of dry weight (DW). The proximate composition, which includes moisture, crude protein, crude fat, and ash, was evaluated according to the method of the Association of Official Analytical Chemists (AOAC) 2019 [25] and the method reported by Wunjuntuk et al. [26]. Moisture and ash contents were determined using the AOAC 952.08 and the 930.30 and 945.46 methods, respectively. Crude protein content was estimated by the Kjeldahl method (AOAC 992.23 method) and calculated from total nitrogen multiplied by 6.25. Crude fat was estimated by the Soxhlet extract method, using petroleum ether (AOAC 948.15 and 945.16 methods). Total carbohydrate was calculated by subtracting the percentage of moisture, protein, fat, and ash from 100%. The energy estimation was determined by multiplying the protein, carbohydrate, and fat contents by 4 and 9 kcal, respectively. Furthermore, vitamin and mineral contents, including thiamine (AOAC 942.23 method), niacin (AOAC 961.14 method), folate (AOAC 960.46 method), zinc (AOAC 984.27 method), iron (AOAC 984.27 method), magnesium (AOAC 984.27 method), calcium (AOAC 985.35 method), and potassium (AOAC 985.35 method), were determined using the AOAC method 2019. Phosphorus was determined by the method reported by Kolthoff et al. [27].

### 2.3. Determination of Dietary Fiber

The total dietary fiber (TDF)—insoluble and soluble dietary fiber (IDF and SDF)—content of DRB was assessed by an enzymatic–gravimetric method, following AOAC Method 991.43 [25] and the method of Wunjuntuk et al. [26].

### 2.4. Determination of Phytic Acid

Phytic acid content was detected with a phytic acid (phytase)/total phosphorus assay kit (K-PHYT, Megazyme International, Wicklow, Ireland), according to the manufacturer’s instructions.

### 2.5. Determination of Total Phenolic Content (TPC)

DRB (3 g) was extracted with 50% N, N-dimethylformamide (DMF; 25 mL) in a water bath at room temperature for 18 h. The extracted solution was centrifuged (9000× *g*/4 °C/15 min), and the supernatant was collected for TPC analysis. A quantity of 25 μL of the extract was mixed with Folin–Ciocalteu reagent (125 μL 10% (*v*/*v*)) and aqueous sodium hydroxide solution (100 μL of 0.5 M) in a 96-well microplate, and incubated in the dark at room temperature for 15 min. The absorbance of the mixture was measured within 15 min using a microplate reader at 750 nm. Gallic acid was used as a standard by diluting it with 50% DMF to 10–100 µg/mL. TPC was expressed in milligram gallic acid equivalents per 100 g DRB (mg GAE/100 g DRB).

### 2.6. Determination of Phenolic Acid Profiles Using a High-Performance Liquid Chromatograph (HPLC)

The extraction was performed according to the method reported in [28], with certain modifications. Briefly, 1 g of DRB was extracted with methanol (80%) at a ratio of 1:10 (*w*/*v*) for 2 h and centrifuged at 4200× *g* for 10 min. The supernatant was dried at 35 °C by a rotary vacuum evaporator and reconstituted to 1.5 mL with ethyl acetate. The residue remaining from the first extraction was washed with 40 mL of distilled water and dried in a Buchner funnel with a vacuum pump. Then, the dried residue was further hydrolyzed with 2M NaOH in a 1:50 (*w*/*v*) ratio for 4 h. Later, the pH of the mixture was adjusted to 2 using 6N HCl, followed by extraction repeated four times with ethyl acetate (4 × 40 mL). The combined ethyl acetate fractions were evaporated and reconstituted to 1.5 mL with ethyl acetate. Both extractions were mixed and filtered with a 0.2 µm polytetrafluoroethylene (PTFE) membrane (Millipore Corp., Cork, Ireland). Then, the filtered sample was injected into a high-performance liquid chromatograph, according to the protocol reported by Sombutsuwan et al. [29]. The phenolic acid profiles in DRB were identified by comparisons of retention times and UV spectra with phenolic acid standards, including 4-hydroxybenzoic acid, vanillic acid, syringic acid, p-coumaric acid, ferulic acid, sinapic acid, and caffeic acid. UV absorbance was monitored at a wavelength of 257 nm for 4-hydroxybenzoic acid and vanillic acid, 271 nm for syringic acid, 300 nm for p-coumaric acid, and 320 nm for ferulic acid, sinapic acid, and caffeic acid. The profiles were quantified by comparing their peak areas with standard calibration curves.

### 2.7. Animal Experiment and Sample Collection

Male Wistar rats (age: four weeks, weight: 90–100 g) were purchased from Nomura Siam International Co., Ltd. Bangkok, Thailand. Firstly, the rats were acclimatized and housed in individually ventilated cages (IVCs) (2 rats/cage) under controlled room conditions (23 ± 1 °C and 50 ± 10% humidity on a 12 h light/dark cycle) for one week before the experimental work. The procedures were approved by the Siriraj Animal Care and Use Committee, Mahidol University (COA no. 004/2562).

**Figure 1:**
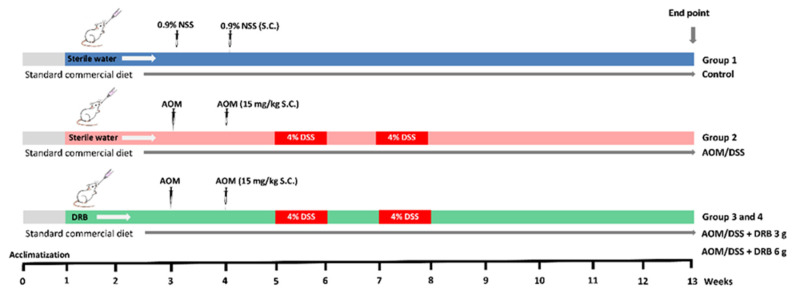
Scheme of AOM/DSS-induced colitis-associated CRC rat model and experimental design. AOM, azoxymethane; DSS, dextran sodium sulfate; DRB, defatted rice bran; NSS, normal saline solution; S.C., subcutaneous injection.

The study was conducted on four groups of rats with randomization, and each group consisted of 10 rats: (1) control, (2) induced AOM/DSS, (3) induced AOM/DSS + DRB 3 g/kg body weight (BW), and (4) induced AOM/DSS + DRB 6 g/kg BW. All rats had free access to a standard commercial diet and water. A schematic representation of the experimental study is shown in Figure 1. After a one-week acclimatization period, groups 3 and 4 were administered with DRB dosages of 3 and 6 g/kg BW by gavage feeding daily throughout the study. The dosing of DRB followed a previous study that reported an administered dose of rice bran of 30 g per day in healthy adults [30] and healthy survivors of CRC [31]. The human dose (mg/kg BW) was then converted to an animal equivalent dose (mg/kg BW) based on the ratios of human and rat body surface areas to body weights of 60 kg for humans and 200 g for rats [32]. After two weeks, groups 2, 3, and 4 received a carcinogenic treatment by subcutaneous injection of AOM (Sigma-Aldrich Pte. Ltd., Singapore; dose: 15 mg/kg BW) once weekly for 2 weeks. One week after the AOM injection, the treatment was followed by 2 cycles of DSS (molecular weight: 36–50 kDa; TdB Consultancy, Uppsala, Sweden) administration. Each cycle included 1 week of 4% (*w*/*v*) DSS in drinking water followed by 1 week of recovery with regular water.

Weight, food intake, and clinical signs of disease in the rats were recorded and monitored daily throughout the experiment. At the end point of the experiment (10 weeks after the first AOM injection), all the rats were euthanized by CO_2_ asphyxiation. The colons from the part of the cecum to the anus were immediately removed and placed on ice-cold plates, longitudinally opened, and carefully washed with cold phosphate-buffered saline solution (PBS). Excess fluid was removed from the colons. Subsequently, the colons were photographed and their weights (g) and lengths (cm) were measured. Then, colon specimens of 5 rats per group were fixed in 10% neutral phosphate-buffered formalin for histopathological and immunohistochemical evaluation, and the remaining specimens were stored at –80 °C for ELISA and Western blot analysis.

### 2.8. Clinical Assessment

The disease activity index (DAI) was used to assess the severity of colitis. It was calculated by scoring three parameters on a scale of 0 to 4, including percentage of weight loss, stool consistency, and rectal bleeding (Table 1). The DAI total score ranged from 0 (no clinical symptoms) to 12 (severe colitis). In addition, colitis was assessed by measuring the weight and length of the colon from the ileocecal junction to the anus. The weight per unit length of the colon was calculated as the colon’s relative weight and length ratio (g/cm).

### 2.9. Quantification of ACFs

Each colon tissue specimen was sectioned into proximal, middle, and distal parts. The sections were fixed flat between filter papers in 10% buffered formalin for at least 24 h. The fixed colonic sections were stained with 0.2% methylene blue for 5 min and placed on slides with the mucosal side up. ACFs were examined under a light microscope at 40× magnification. Criteria that distinguished ACFs from normal crypts included larger crypts with polymorphic shape, irregular and dilated lumen openings, thicker epithelial linings, and pericryptal zones [33]. The numbers of ACFs throughout the colons and the numbers of crypts included in each ACF were counted and recorded.

### 2.10. Quantification of Tumors and Assessment

Tumors were grossly examined for location, including the proximal and distal colon, and counted to assess tumor incidence, distribution, and tumor multiplicity. Tumor incidence was the percentage of total rats with tumors (adenomas and adenocarcinomas), while tumor multiplicity was the average number of tumors per tumor-bearing rat, representing the number of tumors per rat. A Vernier caliper was used to determine the volume, length, width, and depth of each tumor. Tumor volume was determined using the following equation [34]:

(1) $$V\;({\mathrm{cm}}^{3})=\mathrm{length}\;\times \;\mathrm{width}\;\times \;\mathrm{depth}\;\times \;\frac{\pi }{6}$$

### 2.11. Histopathological Examination

Colon tissues were routinely processed for histological analysis. Initially, each tissue specimen was embedded in paraffin, sectioned at 4 µm intervals, and stained with haematoxylin and eosin (H&E) for light microscopic examination. Out of the ten regions of the sections, three sections per rat were randomly chosen and identified as ACFs [20], adenomas, and adenocarcinomas [35]. The proportion of ACFs with hyperplasia, low-grade dysplasia, and high-grade dysplasia was calculated as a percentage per region, and adenomas and adenocarcinomas were calculated as percentages per total tumors. Two independent pathologists examined the tissue sections blindly, and the third pathologist resolved discrepancies between these two examiners until a consensus was reached.

In addition, inflammation of the colon was assessed using two criteria to detect the infiltration of inflammatory cells [36]. Firstly, the severity of leukocyte density in the lamina propria was assessed and graded according to five levels: 0, normal; 1, mild, <10%; 2, mild to moderate, 10−25%; 3, moderate, 26−50%; and 4, severe, >51%. Secondly, the expansion of leukocytes was divided into four levels: 0, normal, no extent; 1, mild, extent to the mucosa; 2, moderate, extent to the mucosa and submucosa; and 3, severe, transmural extent. A mean score was calculated for ten fields per rat at 40× magnification, representing the infiltration of inflammatory cells in each experimental group.

### 2.12. Enzyme-Linked Immunosorbent Assay (ELISA) Analysis

Briefly, 150 mg of colon tissue was cut into small pieces and homogenized in cold PBS, then centrifuged at 10,000× *g* for 15 min at 4 °C. The supernatant was collected to quantify the cytokines IL-6 and IL-10 by sandwich ELISA (900-M86 and 900-M53, Peprotech, Cranbury, NJ, USA), according to the manufacturer’s protocol.

### 2.13. Western Blot Analysis

Colon tissues were lysed in a cold lysis buffer (Abcam, Cambridge, UK) with protease and phosphatase inhibitor (Sigma-Aldrich Pte. Ltd., Singapore) and centrifuged at 10,000× *g* for 15 min at 4 °C. Protein quantification was performed using BCA protein assay reagent (Pierce, USA). Protein lysate (40 μg) was separated first by 10% SDS-PAGE and then transferred onto nitrocellulose membranes (Bio-Rad Laboratories Ltd., Feldkirchen, Germany). The membranes were blocked with 5% skim milk in TBST (0.1% Tween 20 in Tris-buffered saline) for 1 h and then probed with anti-COX-2 (13120S) and anti-phospho-p65 NF-κB (3033S) antibodies (Cell Signaling Technology, Danvers, Massachusetts, USA) at a 1:1000 dilution in TBST at 4 °C overnight. Afterward, the membranes were rinsed with 1X TBST and incubated with HRP-conjugated secondary antibodies (7074S, Cell Signaling Technology, Danvers, MA, USA) at a 1:2000 dilution in TBST for 2 h. For visualization, protein bands were detected by enhanced chemiluminescence (ECL) at a dilution of 1:1 (Bio-Rad Laboratories Ltd., Feldkirchen, Germany). The intensity of the protein bands was quantified using ImageJ software version 1.52a. COX-2 and NF-κB expression levels were calculated and reported as relative β-actin expression.

### 2.14. Immunohistochemical Analysis

The protein expression of β-catenin, cyclin D1, and c-Myc in the colon tumors was detected by immunohistochemistry. The paraffin-embedded sections were deparaffinized before heating with 10 mM citrate buffer (pH 6.0) at 90–95 °C for 15 min. The sections were incubated with 3% hydrogen peroxide for 15 min and blocked with normal goat serum for 2.30 h. Furthermore, each section was sequentially incubated with anti-β-catenin (ab32572), anti-cyclin D1 (ab16663), and anti-c-Myc (ab32072) antibodies (Abcam, Cambridge, UK) at a dilution of 1:50 of each at 4 °C overnight and with a biotinylated secondary antibody (VECTASTAIN^®^ ABC-HRP kit) and 3,3′-diaminobenzidine (Vector^®^ DAB peroxidase substrate) (purchased from Vector Laboratories, Burlingame, CA, USA), following the manufacturers’ protocols. Then, the slides were counterstained with Mayer’s hematoxylin.

The immunohistochemically stained sections were observed under a light microscope. Five different fields of each section per rat were randomly photographed. The expression of β-catenin, cyclin D1, and c-Myc in the colon tumors was evaluated based on the intensity of staining and cellular localization compared to the background control, according to the criteria described by Serafino et al. [37].

### 2.15. Statistical Analysis

All data were expressed as means ± standard errors of the means (SEMs). Statistical software (SPSS Inc. version 17.0, Chicago, IL, USA) was used to analyze the statistically significant differences in this experimental study. Statistical analysis was performed using one-way ANOVA followed by post hoc multiple comparisons and analyses using Tukey’s test to compare all experimental groups. Statistically significant differences were considered at a *p*-value of less than 0.05.

## 3. Results

### 3.1. Proximate Analysis and Nutrient Composition

The proximate analysis of DRB is shown in Table 2. DRB/100 g dry weight (DW) provides 348.83 ± 3.88 kcal energy, 66.12 ± 0.68 g carbohydrate, 16.06 ± 0.47 g protein, 2.23 ± 0.49 g fat, 9.76 ± 0.21 g ash, and 5.82 ± 0.27 g moisture. DRB contents (presented as mg per 100 g DW) include thiamine (2.63 ± 0.21 mg), niacin (30.31 ± 7.20 mg), folate (150.0 ± 11.14 mg), zinc (6.01 ± 0.38 mg), iron (11.60 ± 1.65 g), calcium (46.03 ± 1.28 mg), potassium (1574.61 ± 20.61 mg), phosphorus (2176.88 ± 15.25 mg), and magnesium (2217.56 ± 333.16 mg), as shown in Table 3, Supplementary S1 and S2.

### 3.2. Dietary Fiber Content

The dietary fiber contents of DRB are shown in Table 4. The total dietary fiber (TDF), insoluble dietary fiber (IDF), and soluble dietary fiber (SDF) contents in DRB were determined as 24.00 ± 0.19, 20.68 ± 0.26, and 3.32 ± 0.07 g, respectively.

### 3.3. Phytochemical Composition

DRB had high phytic acid contents of 6.94 ± 0.81 mg/100 g DRB. Total phenolic content and phenolic acid profiles in DRB are presented in Table 5. The total phenolic content of 477.55 ± 18.98 mg GAE/100 g DRB was estimated by Folin–Ciocalteu assay. Seven phenolic acids were analyzed in DRB by HPLC. The most abundant was ferulic acid (199.57 ± 5.40 mg), followed by *p*-coumaric acid (61.61 ± 3.54 mg), sinapic acid (2.54 ± 0.48 mg), syringic acid (1.04 ± 0.06 mg), and vanillic acid (1.03 ± 0.17 mg). Caffeic acid and 4-hydroxybenzoic acid were not detected.

### 3.4. Body Weight and Food Intake

**Figure 2:**
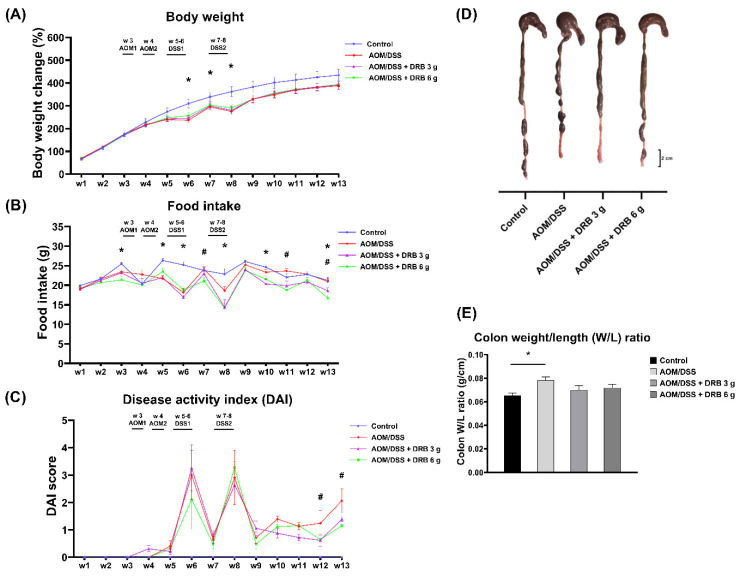
Effect of DRB on: (**A**) body weight changes; (**B**) food intake; (**C**) disease activity index (DAI) during colon carcinogenesis in the AOM/DSS-induced colitis-associated colorectal cancer rat model. (**D**) Representative images of the morphological changes in the colon. The black bar represents 2 cm. (**E**) Weight–length ratios of the colons in each experimental group. All data are expressed as means ± SEMs (*n* = 10). Asterisks (*) and number signs (#) indicate statistically significant results for the control and AOM/DSS groups (*p* < 0.05), respectively. The statistical analysis used one-way ANOVA followed by Tukey’s test. AOM, azoxymethane; DSS, dextran sodium sulfate; DRB, defatted rice bran.

Body weight and food intake were analyzed and recorded for all the rats. Body weight increased for all rats throughout the experiment. Compared to the control group, the percentage changes in the body weights of the rats administered AOM/DSS, AOM/DSS + DRB 3 g, and AOM/DSS + DRB 6 g were smaller and showed remarkable differences (*p* < 0.05) during the DSS-induced period (Figure 2A). However, no significant change was observed in the body weights of rats supplemented with DRB compared to the AOM/DSS group. In addition, the food intake of rats decreased in the AOM/DSS combined DRB-supplemented groups compared to the control and AOM/DSS groups at weeks 7, 11, and 13 (Figure 2B).

### 3.5. DRB Supplementation Reduced DAIs and Colon Weight–Length Ratios in AOM/DSS-Induced Colitis-Associated CRC Rats

DAI parameters, such as percentage weight loss, stool consistency, and rectal bleeding, were used to evaluate the clinical symptoms of colitis and CRC. No clinical symptoms were observed in normal rats. In contrast, signs of colitis, such as diarrhea and rectal bleeding, gradually appeared in rats in the AOM/DSS-induced groups during the first cycle of DSS administration throughout the experimental period. During the second cycle of the same DSS administration, the rats showed the highest DAI scores due to severe diarrhea and rectal bleeding. The DAI scores decreased in the recovery phase (without DSS administration) and increased till the completion of the study. Interestingly, DAI scores were lower in rats in both DRB-supplemented groups (*p* < 0.05) than in rats treated with AOM/DSS alone (Figure 2C). In addition, ratios of colon weight to length, a marker of colitis-associated colonic edema, were also examined. Compared to the control group, the AOM/DSS group showed thickening and shortening of the colon and a significant increase in colonic weight–length ratio (Figure 2D and E), whereas colonic weight–length ratio decreased in the AOM/DSS + DRB 3 g group and the AOM/DSS + DRB 6 g group compared to the AOM/DSS group. However, there was no significant difference in colon weight–length ratios between the AOM/DSS + DRB 3 g and the AOM/DSS + DRB 6 g groups. These results suggest that DRB supplementation attenuated the clinical symptoms of colitis and colonic edema in the AOM/DSS-induced colitis-associated CRC rat model.

### 3.6. DRB Supplementation Suppressed the Progression of ACF Development in AOM/DSS-Induced Colitis-Associated CRC Rats

**Figure 3:**
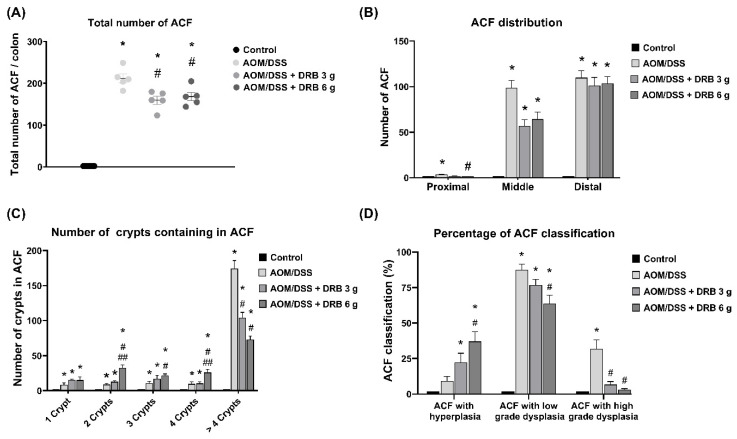
Effect of DRB on: (**A**) total number of ACFs per colon; (**B**) ACF distribution; (**C**) number of crypts in each ACF; (**D**) percentage of ACFs classification. All data are presented as means ± SEMs (*n* = 5). Bars with asterisks (*) and number signs (#) indicate statistically significant results for the control and AOM/DSS groups, respectively (*p* < 0.05). Double number signs (##) indicate statistically significant differences between the AOM/DSS + DRB 3 g group and the AOM/DSS + DRB 6 g group (*p* < 0.05). The statistical analysis used one-way ANOVA followed by Tukey’s test. AOM, azoxymethane; DSS, dextran sodium sulphate; DRB, defatted rice bran.

ACFs were absent from the colons of normal rats. However, all rats in the AOM/DSS-induced groups had ACFs in the colon (100% of ACF incidence). From the data, it was concluded that the rats in the AOM/DSS group had the highest numbers of ACFs—an average of 210 total ACFs/colon (Figure 3A). The total number of ACFs/colon was significantly reduced in the AOM/DSS + DRB 3 g (mean 160 ACFs/colon) and AOM/DSS + DRB 6 g (mean 170 ACFs/colon) groups compared to the control group (*p* < 0.05). The distribution of ACFs was mainly marked in the distal region, followed by the middle and proximal parts of the colon (Figure 3B). The numbers of crypts in ACFs are shown in Figure 3C. Small (1−2 crypts/ACF) and medium (3−4 crypts/ACF) ACFs were found more frequently in the DRB-supplemented groups than in the AOM/DSS group. Conversely, the number of large ACFs (>4 crypts/ACF) was markedly reduced in the DRB-supplemented groups compared to the AOM/DSS group (*p* < 0.05). However, there was no significant difference in the number of large ACFs between the two groups (AOM/DSS + DRB 3 g and AOM/DSS + DRB 6 g). In addition, the histopathology of the ACFs examined by H&E staining was categorized into three grades: ACFs with hyperplasia, ACFs with low-grade dysplasia, and ACFs with high-grade dysplasia. The proportions of ACFs with hyperplasia were higher in the DRB-supplemented groups than in the AOM/DSS group (Figure 3D). In contrast, the proportion of ACFs with low-grade dysplasia and high-grade dysplasia was significantly higher in the AOM/DSS group than in the DRB-supplemented groups (*p* < 0.05). Altogether, these results suggest that DRB supplementation can suppress and attenuate the progression of ACF development in the AOM/DSS-induced colitis-associated CRC rat model.

### 3.7. DRB Supplementation Alleviated the Progression of Tumor Development in AOM/DSS-Induced Colitis-Associated CRC Rats

**Figure 4:**
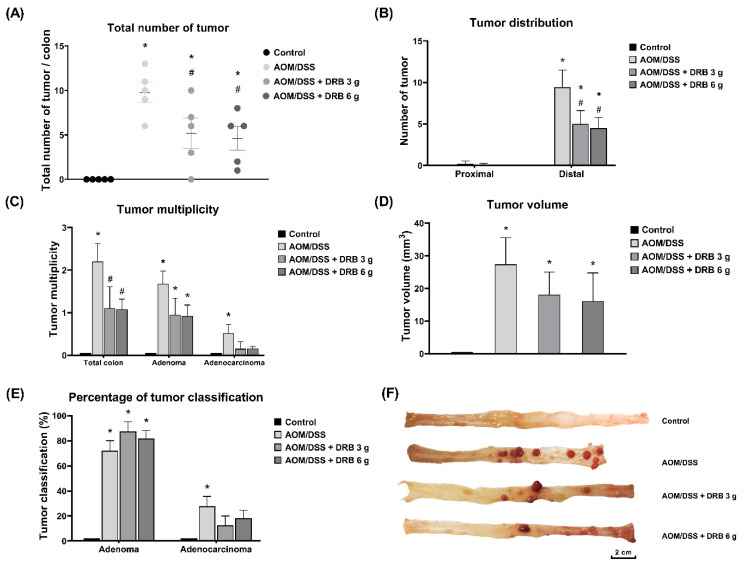
Effect of DRB on: (**A**) tumor incidence; (**B**) tumor distribution; (**C**) tumor multiplicity; (**D**) tumor volume; (**E**) percentage of tumor classification. (**F**) Representative images of the colonic tumors in each experimental group. The black bar represents 2 cm. All data are presented as means ± SEMs (*n* = 5). Bars with asterisks (*) and number signs (#) indicate statistically significant results for the control and AOM/DSS groups, respectively (*p* < 0.05). The statistical analysis used one-way ANOVA followed by Tukey’s test. AOM, azoxymethane; DSS, dextran sodium sulphate; DRB, defatted rice bran.

There was an absence of tumors in the colons of the normal rats. The AOM/DSS and AOM/DSS + DRB 6 g groups evidenced 100% tumor presence, while the AOM/DSS + DRB 3 g group revealed 90% presence. On the whole, the total number of colon tumors was highest in the AOM/DSS group, with about ten tumors per colon (Figure 4A,F). Contrarily, the total number of tumors per colon was significantly lower in the AOM/DSS + DRB 3 g group (mean: five tumors/colon) and in the AOM/DSS + DRB 6 g group (mean: 4.5 tumors/colon) than in the AOM/DSS group (*p* < 0.05). Nevertheless, the total numbers of tumors per colon in the two DRB-supplemented groups showed no statistically significant differences. Mostly, the tumors were distributed in the distal part of the colon (Figure 4B). In contrast to the AOM/DSS group, the numbers of tumors in the AOM/DSS + DRB 3 g and AOM/DSS + DRB 6 g groups were found to be significantly lower (*p* < 0.05). The multiplication rates of colon tumors (Figure 4C) in the AOM/DSS + DRB 3 g and AOM/DSS + DRB 6 g groups were considerably lower than in the group of rats treated with AOM/DSS alone (*p* < 0.05). In addition, the two DRB-supplemented groups had lower adenoma and adenocarcinoma tumor multiplicities than the AOM/DSS group. The two DRB-supplemented groups showed smaller tumor sizes than the AOM/DSS group (Figure 4D).

Colon tumor histopathology was determined according to the percentages of adenoma and adenocarcinoma formation. An adenoma is a benign tumor, while an adenocarcinoma is a malignant tumor in the colon. The DRB-supplemented groups of rats showed the highest percentages of adenomas as compared with the AOM/DSS group (Figure 4E). The percentages of adenocarcinomas in the two groups of DRB-supplemented rats were smaller than the percentage in the AOM/DSS group. These results suggested that the supplementation with DRB can attenuate the progression of tumor development to carcinoma in the AOM/DSS-induced colitis-associated CRC rat model.

### 3.8. DRB Supplementation Decreased Inflammatory Cell Infiltration in AOM/DSS-Induced Colitis-Associated CRC Rats

**Figure 5:**
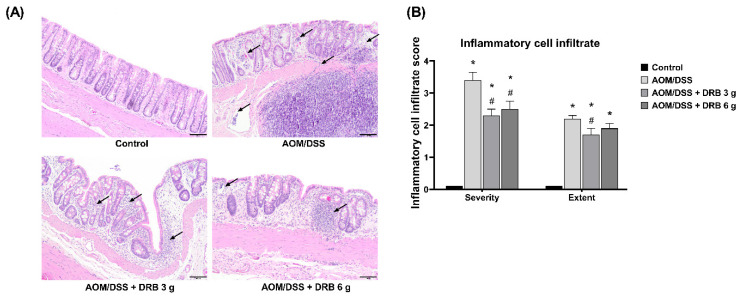
Effect of DRB on inflammatory cell infiltration. (**A**) Representative images from the histopathological analysis of colon tissue sections stained with hematoxylin/eosin (H&E) under a light microscope at 100× magnification. Scale bars represent 100 µm. Black arrows indicate inflammatory cell infiltrates in colonic tissue. (**B**) Inflammatory cell infiltration score. All data are presented as means ± SEMs (*n* = 5). Bars with asterisks (*) and number signs (#) indicate statistically significant results for the control and AOM/DSS groups, respectively (*p* < 0.05). The statistical analysis used one-way ANOVA followed by Tukey’s test. AOM, azoxymethane; DSS, dextran sodium sulphate; DRB, defatted rice bran.

Histopathology of inflammatory cell infiltration in colonic tissue sections was evaluated to confirm colitis (Figure 5A). The AOM/DSS-induced groups showed high leukocyte densities in the lamina propria and submucosa. In addition, the AOM/DSS group demonstrated moderate to severe inflammatory cell infiltration in the mucosa and infiltration moderately extended to the submucosa, while the AOM/DSS + DRB 3 g and AOM/DSS + DRB 6 g groups showed moderate inflammatory cell infiltration in the mucosa and infiltration slightly extended to the submucosa. The AOM/DSS group had the highest scores for the severity and extent of inflammatory cell infiltration (Figure 5B), while the DRB- supplemented groups of rats showed significantly lower scores (*p* < 0.05). However, no significant differences in inflammatory cell infiltration scores were observed between the AOM/DSS + DRB 3 g group and the AOM/DSS + DRB 6 g group. These results indicate that DRB supplementation can decrease inflammatory cell infiltration in the lamina propria and submucosa in the AOM/DSS-induced colitis-associated CRC rat model.

### 3.9. DRB Supplementation Inhibited Inflammation in AOM/DSS-Induced Colitis-Associated CRC Rats

**Figure 6:**
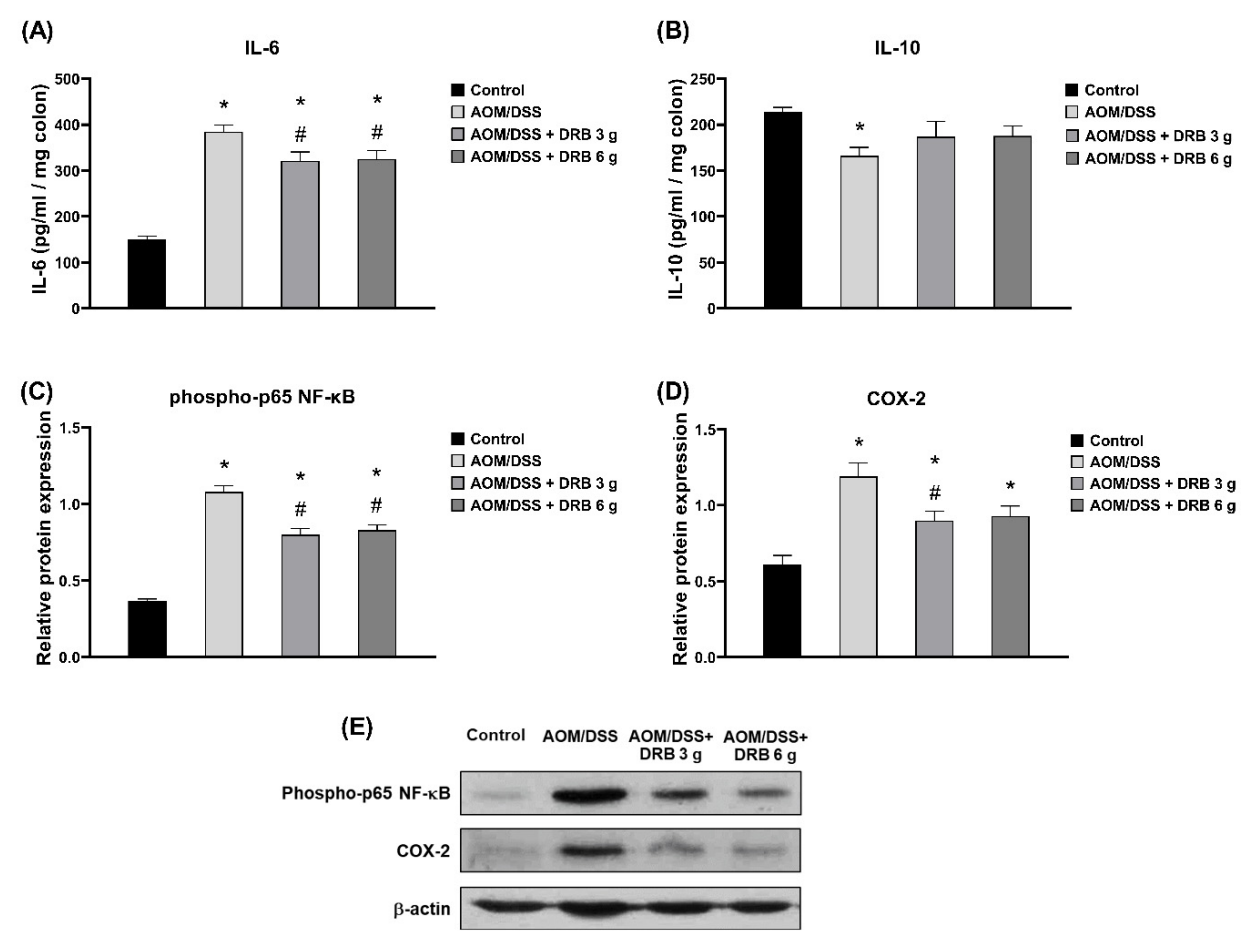
Effect of DRB on inflammation markers: (**A**) pro-inflammatory cytokine levels (IL-6) and (**B**) anti-inflammatory cytokine levels (IL-10), as determined by ELISA analysis. Protein expression levels: (**C**) phospho-p65 NF-κB and (**D**) COX-2, as determined by Western blot analysis. (**E**) Representative images of protein bands in each experimental group. The band intensities were measured with Image J software version 1.52a, Bethesda, Maryland, USA and normalized to β-actin. All data are presented as means ± SEMs (*n* = 5). Bars with asterisks (*) and number signs (#) indicate statistically significant results for the control and AOM/DSS group, respectively (*p* < 0.05). The statistical analysis used one-way ANOVA followed by Tukey’s test. AOM, azoxymethane; DSS, dextran sodium sulphate; DRB, defatted rice bran.

The AOM/DSS group showed an increase in IL-6 colonic tissue pro-inflammatory cytokines, whereas the levels of IL-10 anti-inflammatory cytokines decreased. The two groups of DRB-supplemented rats showed significant inhibition of IL-6 (*p* < 0.05) and raised IL-10 levels compared to the AOM/DSS group (Figure 6A,B). In addition, the results for the expression of inflammatory proteins are presented in Figure 6C−E. The AOM/DSS group showed an increase in phospho-p65 NF-κB and COX-2 expression, while the two groups of DRB-supplemented rats showed significant suppression of phospho-p65 NF-κB and COX-2 expression (*p* < 0.05). From the analysis of the results, we concluded that the supplementation with DRB can inhibit pro-inflammatory cytokines (IL-6) and induce anti-inflammatory cytokine (IL-10) secretion in the colonic tissue. Moreover, DRB supplementation can also suppress phospho-p65 NF-κB and COX-2 expression in the AOM/DSS-induced colitis-associated CRC rat model.

### 3.10. DRB Supplementation Reduced Expression of Cancer Cell Proliferation Proteins in AOM/DSS-Induced Colitis-Associated CRC Rats

**Figure 7:**
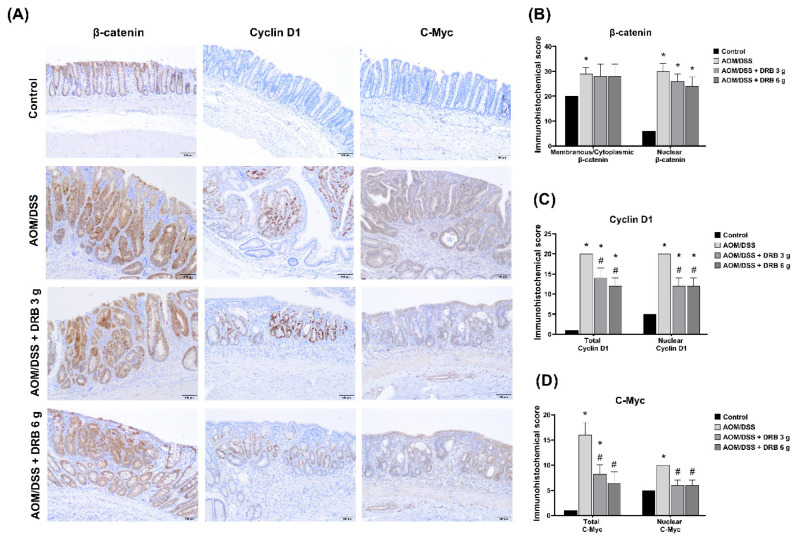
Effect of DRB on cancer cell proliferation protein expression in colonic tissue. (**A**) Representative images of the immunohistochemical colonic tissue sections stained with β-catenin, cyclin D1, and c-Myc under a light microscope at 100X magnification. Scale bars represent 100 µm. (**B**–**D**) Immunohistochemical scores for β-catenin, cyclin D1, and c-Myc. All data are expressed as means ± SEMs (*n* = 5). Bars with asterisks (*) and number signs (#) indicate statistically significant results for the control and AOM/DSS groups, respectively (*p* < 0.05). The statistical analysis used one-way ANOVA followed by Tukey’s test. AOM, azoxymethane; DSS, dextran sodium sulphate; DRB, defatted rice bran.

The accumulation of nuclear β-catenin is associated with CRC progression through the Wnt/β-catenin signaling pathway. Therefore, we investigated the effect of DRB on molecular markers, including β-catenin, cyclin D1, and c-Myc, which involved the assessment of proliferative cell regulation in the Wnt/β-catenin signaling pathway by immunohistochemistry analysis. The intensities and percentages of positive cell staining of β-catenin, cyclin D1, and c-Myc in colonic tissues are shown in Figure 7A. The expression of β-catenin was observed in both the cell membranes and nuclei of colonic tissues. In the control group, normal colonic mucosa showed β-catenin staining in the cell membranes (Figure 7B). On the contrary, the AOM/DSS-induced groups revealed increased β-catenin positive cell staining expression in both cell membranes and nuclei as compared to the control group. The highest scores for nuclear β-catenin expression were observed in the AOM/DSS group, whereas the DRB-supplemented groups displayed lower scores. The expression of cyclin D1 and c-Myc markers was located in the nucleus (Figure 7A). Total cyclin D1 and c-Myc expression (Figure 7C,D) was determined by staining intensity, and nuclear cyclin D1 and c-Myc expression was determined by percentage of positive cell staining. In the control group, the staining intensity for cyclin D1 and c-Myc was similar to the background staining. The percentage of positive cell staining was found to be <10%. The AOM/DSS group showed intense staining and a high rate of positive cells (>50%). Remarkably, both DRB-supplemented groups showed significantly decreased staining intensities and percentages of positive cell staining for cyclin D1 and c-Myc compared to the AOM/DSS alone group (*p* < 0.05). The tested results indicated that the administration of DRB attenuated β-catenin, cyclin D1, and c-Myc expression in AOM/DSS-induced rats, which was correlated with reductions in numbers of ACFs and tumor formation in the colon.

## 4. Discussion

Food consumption behavior has undergone considerable change, and diet is one of the crucial factors that accelerates CRC through several mechanistic pathways. Natural dietary compounds can prevent CRC. In our study, we considered that people ought to focus on natural healthy diets. Therefore, we looked for a natural nutritional product that would be beneficial for CRC prevention and enhance healthy food choices. Hence, in this study, we utilized DRB, a by-product obtained after rice milling and oil extraction, for the chemoprevention of CRC. However, research on DRB as a chemopreventive agent is still in its infancy. Accordingly, in this study we attempted to determine the bioactive compounds in DRB and investigate its chemopreventive effects, including anti-inflammatory, anti-proliferative, and anti-cancer effects, in an AOM/DSS-induced colitis-associated CRC model.

The AOM/DSS-induced colitis-associated CRC model is widely used in the experimental study of animals to investigate chemopreventive effects through the mimicry of human sporadic colon cancer clinical symptoms and histopathological and molecular alterations [38,39]. In this model, two injections of AOM and its combination with two cyclic administrations of DSS in drinking water were employed to induce chronic inflammation, dysplasia, and cancer development in the colon. AOM is a colonic carcinogen; it metabolizes, causing CRC through WNT/β-catenin pathway activation to promote cell proliferation, differentiation, and migration [40]. In comparison, DSS induces colonic inflammation by directly damaging the colon’s epithelial lining and affecting the mucosal barrier’s integrity [41,42]. Prolonged colonic inflammation leads to CRC development.

In the present study, we evaluated the chemopreventive effects of DRB in an AOM/DSS-induced colitis-associated CRC rat model. Our results showed that the percentage body weight changes and food intakes of rats in the AOM/DSS-induced groups were lower than those of normal rats. The clinical signs of CRC might be responsible for this effect. Moreover, intake of DRB doses of 3 and 6 *g/kg* BW per day was accompanied by a rapid decrease in food intake due to the high dietary fiber content of DRB. In addition, soluble fiber can enhance the viscosity and water-holding capacity of digesta and induces gel formation [43]. All these properties can delay gastric emptying, leading to satiation [44]. However, these results cannot sufficiently explain whether a decrease in food intake and body weight in both the DRB-supplemented groups was the effect of DRB or a manifestation of clinical signs.

Chronic intestinal inflammation is an initial step in CRC development. Several studies have demonstrated that patients with inflammatory bowel disease (IBD) have an approximately two- to three-fold increased risk of CRC compared to healthy populations [45]. Therefore, controlling inflammation will block the development of CRC. As a significant finding of this study, it was concluded that DRB supplementation reduced chronic inflammation in the AOM/DSS-induced colitis-associated CRC model. Furthermore, we evaluated the clinical symptoms of colitis by DAI, including body weight loss, stool consistency, and rectal bleeding, in a subjective assessment of chronic colitis disease severity. Analysis of the results showed that DRB supplementation reduced DAI scores, indicating that DRB alleviates the clinical symptoms of colitis. In addition, colonic edema indicates the severity of chronic colitis through the measurement of change in colon weight–length ratio. The characteristics of colitis are thickening and shortening of the colon and increased colon weight–length ratio. In our results, both DRB supplementation doses slightly affected colon weight–length ratios. However, DAI and colon weight–length ratio are clinical symptoms that can be assessed visually. Therefore, it is impossible to evaluate comprehensively chronic colitis disease severity using only clinical signs.

In addition, chronic colitis is associated with the activation of NF-κB. The inflammatory cells are recruited and accumulate in the local tissue during the inflammation process. Inflammatory cytokines (such as TNF-α, IL-1 β, and IL-6) are released from inflammatory cells and activate the NF-κB signaling pathway—an essential mediator of inflammatory responses. NF-κB is an inducible transcription factor that can induce the expression of pro-inflammatory cytokine genes (such as TNF-α, IL-1β, IL-6, and IL-8) and inflammatory enzymes (such as COX-2 and i-NOS) [46]. In addition, NF-κB can regulate cell proliferation (such as cyclin D1 and c-Myc), survival, and differentiation [47,48]. Herein, we confirmed the effects of DRB with respect to anti-inflammation potential by histopathological characterization and observation of inflammatory cytokine production in the colon tissues. Severe inflammatory cell infiltration and increased inflammatory cytokine production in colon tissue also affected CRC development in the AOM/DSS-induced colitis-associated CRC model. Our results showed that DRB supplementation reduced inflammatory cell infiltration in colon tissue and decreased pro-inflammatory cytokine (IL-6) production and inflammatory mediator (NF-κB and COX-2) expression. At the same time, anti-inflammatory cytokine (IL-10) production increased with DRB supplementation. The results demonstrated that DRB supplementation not only alleviated the pathological symptoms of colitis-associated CRC but also reduced levels of pro-inflammatory cytokine production and inflammatory mediator expression. In addition, histopathological evaluation confirmed that DRB supplementation decreased inflammatory cell infiltration in colon tissue, which may be related to the reduction in pro-inflammatory cytokine production and inflammatory mediator expression. Thus, our results suggested that DRB has a protective effect against inflammation in AOM/DSS-induced colitis-associated CRC rats by alleviating clinical symptoms, morphological and histopathological changes, and inflammation marker expression.

Excessive cell proliferation due to sustained proliferative signaling is one of the most common characteristics of cancers [49]. Thus, the measurement of cancer cell proliferation is a pivotal point for investigating cancer development and progression. Several signaling pathways involve cell proliferation, such as the Wnt/β-catenin pathway. Chronic intestinal inflammation is recognized to enhance Wnt/β-catenin signaling though inflammatory signaling pathways, including NF-κB, mitogen-activated protein kinase (MAPK), protein kinase B (PKB/AKT), and signal transducer and activator of transcription (STAT) signaling, which further activate cell proliferation and CRC development [50]. Inflammatory cytokines (TNF-α and IL-6) and inflammatory mediators, such as COX-2, are more expressed during inflammation. They are mainly triggered by the NF-κB signaling pathway in epithelial cells, the activation of which increases β-catenin signaling [9]. Increasing evidence suggests that β-catenin translocates into the nucleus and activates cyclin D1 and c-Myc, contributing to CRC development and progression. In the present study, we observed expressions of β-catenin, cyclin D1, and c-Myc to evaluate the proliferative effects of cell-involved WNT/β-catenin signaling pathways. The results demonstrated that the DRB supplementation attenuated expressions of nuclear β-catenin, cyclin D1, and c-Myc in tumors in colon tissue. Therefore, it appears that the DRB supplementation might be responsible for inhibiting β-catenin and TCF/LEF interaction, leading to decreases in β-catenin, cyclin D1, and c-Myc expression in the nucleus, which, given the role of the WNT/β-catenin signaling pathway, may be related to the reductions in the total numbers of ACFs and tumors.

Another important finding from this study suggested that DRB supplementation reduced ACF numbers and tumor formation in the AOM/DSS-induced colitis-associated CRC model. ACFs are preneoplastic markers, usually used to investigate the protective effects of diet interventions in colon carcinogenesis. ACF incidence, ACF distribution, the number of crypts contained in ACFs, and histopathological analysis of ACFs were used to evaluate the chemopreventive effect of DRB on AFC formation. The study also found that all groups of rats induced with AOM/DSS developed ACFs. Therefore, it was established that DRB supplementation did not affect ACF incidence. In rats induced with AOM/DSS, ACFs were mainly found in the distal colon, followed by the middle part, and were negligible in the area of the proximal colon. It was noticed that the DRB supplementation reduced the number of ACFs in the middle colon. However, it did not affect the formation of ACFs in the distal colon. The as-produced large ACFs had the potential to develop into tumors. The numbers of large ACFs were simultaneously reduced in the middle colon by DRB supplementation. However, DRB did not affect small and medium ACFs. These results might be due to the prolonged period of the study. We sacrificed rats ten weeks after the first AOM injection. All these conditions might have been responsible for the excessive production of large ACFs as compared with the small and medium ACFs observed in rats induced with AOM/DSS. Correspondingly, the histopathological analysis of ACFs with hyperplasia in the DRB supplementation groups showed higher ACF numbers than in than the AOM/DSS alone group; however, ACFs with low- and high-grade dysplasia were alleviated in the DRB supplementation groups compared to the AOM/DSS group. The decrease in numbers of large ACFs and dysplasic ACFs implies that DRB might have suppressed the growth of ACFs at the post-initiation or promotion stage of CRC.

Moreover, this study showed that the DRB supplementation at a dose of 3 g/kg BW per day reduced tumor occurrence by 10%. The colon tumors were found mainly in the distal colon, and the DRB supplementation significantly reduced the numbers of tumors in this part. Similarly, it was discovered that the multiplicity and volume of colon tumors were notably lower in the DRB supplementation groups. In addition, the colon tumors were classified as adenomas and adenocarcinomas based on the histopathological changes in the colon crypts. It was also observed that adenomas (benign tumors) were found in the DRB-supplemented group, while adenocarcinomas (malignant tumors) were primarily found in the AOM/DSS alone group, suggesting that supplementation suppresses the growth of colon tumorigenesis. Simultaneously, DRB could delay the developmental process of preneoplastic ACFs and colon tumors.

The gathered information demonstrated that DRB exhibited cancer chemopreventive potential, which might be associated with bioactive compounds present in DRB. DRB has high contents of proteins, vitamin B (thiamin, niacin, and folate), minerals, dietary fiber and phytochemicals, especially phytic acid, ferulic acid, and *p*-coumaric acid. Although several studies have demonstrated the chemopreventive prospects of rice bran in the AOM/DSS-induced colitis-associated CRC model [17], few studies have reported the chemopreventive effects of DRB in this model. In other models, there was evidence to support the chemopreventive effects of DRB. Saisavoey et al. reported that protein hydrolysates of DRB exhibited chemopreventive effects by reducing levels of pro-inflammatory cytokines (IL-6 and TNF-α) and increasing anti-inflammatory cytokines (IL-10) in Raw 264.7 macrophage cells [51]. Similarly, Dokkaew et al. found that defatted sticky rice bran (administration: 500 mg/kg BW/day for 15 weeks) decreased the numbers and sizes of hepatic glutathione S-transferase placental (GST-P) positive foci. In addition, bran was also found to be responsible for reducing numbers of proliferating nuclear antigen-positive hepatocytes and impairing mRNA expression (TNF-α, i-NOS, and NF-κB) involved in the inflammation process during the early stages of hepatocarcinogenesis in diethylnitrosamine (DEN)-initiated rats [52].

It is known that DRB is a rich source of dietary fiber, protein, vitamins, minerals, and other phytochemicals. Phytic, ferulic, and *p*-coumaric acids were highlighted as the most significant phytochemicals present in DRB. There is evidence to suggest that these phytochemicals have chemopreventive effects. Shafie et al. reported that the administration of phytic acid extracted from rice bran at doses of 0.2% (*w*/*v*), 0.5% (*w*/*v*), and 1.0% (*w*/*v*) via drinking water for 16 weeks reduced numbers of ACFs and tumor incidence. The treatments decreased the expression of β-catenin and COX-2 at protein and mRNA levels in rats with AOM-induced colon carcinogenesis [21]. A similar study demonstrated that administration of phytic acid at doses of 0.25, 0.5, and 1 g/kg BW per day by gavage for 39 weeks decreased tumor incidence and reduced serum levels of pro-inflammatory cytokines (TNF-α, IL-1β, and IL-6) in a 1,2-dimethylhydrazine-induced rat colorectal cancer model. Moreover, the administration of phytic acid improved histopathological changes in colon tumors and reduced damage to intestinal gland structures [22]. Administration of ferulic acid (20 and 40 mg/kg BW) for 14 weeks decreased myeloperoxidase levels, down-regulated mRNA expression of TNF-α, IL-1β, IL-6, i-NOS, and COX-2, up-regulated IL-10 mRNA expression, and restored histopathological aberrations in colon tissues in trinitrobenzene sulfonic acid-induced ulcerative colitis in rats [53]. Sharada et al. found that supplementation with *p*-coumaric acid at doses of 50, 100, and 200 mg/kg BW reduced numbers of ACFs and dysplasic ACFs and polyp incidence in 1,2-dimethylhydrazine (DMH)-induced colonic preneoplastic lesions in rats. Moreover, *p*-coumaric acid can prevent mucin depletion and reduce β-catenin immunoreactivity in colonic crypts [54]. Similarly, in vitro studies have also shown that *p*-coumaric acid inhibited the expression of TNF-α, IL-1β, i-NOS, COX-2, and NF-κB and suppressed the phosphorylation of IκB and ERK1/2 involved in inflammation via the NF-κB and MAPKs signaling pathways in lipopolysaccharide (LPS)-induced inflammatory responses in RAW 264.7 macrophage cells [55]. In addition, dietary fiber has been reported to have chemopreventive effects. Liu et al. found that administration of 1.75 and 3.5 g/kg BW of insoluble dietary fiber extracted from DRB significantly reduced TNF-α, IL-6, and LPS concentrations in serum after 6 weeks of treatment in hyperlipidemic high-fat-diet-fed rats [56]. Moreover, we found that the most abundant mineral in DRB was magnesium. Magnesium plays an important role in many biological processes, including inflammation, DNA replication and repair, cell proliferation, and signaling transduction, all of which are related to carcinogenesis [57]. Previous studies have reported that magnesium deficiency is common in patients with active IBD and in rodent colitis models [58,59,60], and there is evidence showing that magnesium supplementation could reduce colonic inflammation. Trapani et al. found that DSS-treated hypo-magnesium (30 mg/kg Mg) mice exhibited exacerbated colitis. On the other hand, DSS-treated hyper-magnesium (4000 mg/kg Mg) mice had reduced DAI scores and mucosal inflammation compared with hypo-magnesium and control mice at day 12 [61]. In addition, Cui et al. reported that intraperitoneal administration of magnesium isoglycyrrhizinate (5 mg/kg BW) alleviated acute and chronic colitis by reducing immune cell infiltration and suppressing pro-inflammatory cytokine (IL-1β, IL-6, IL-17, IFN-γ, and TNF-α) production in DSS-induced colitis mice [62]. Therefore, the chemopreventive effects of DRB on AOM/DSS-induced colitis-associated CRC might be attributed to the several bioactive compounds present in DRB.

Our study demonstrated that DRB exhibited chemopreventive effects through anti-inflammatory, anti-proliferative, and anti-cancer activities in rats with AOM/DSS-induced colitis-associated CRC. Interestingly, we found that increasing DRB intake (to a dose of 6 mg/kg BW per day) did not enhance anti-inflammation, anti-cell proliferation, or anti-cancer incidence. Therefore, DRB supplementation at doses of 3 mg/kg BW per day is sufficient for chemoprevention in rats with AOM/DSS-induced colitis-associated CRC. The proposed mechanism for DRB’s alleviation of CRC development includes inhibition of inflammatory marker expression via the NF-κB signaling pathway and blocking of the activation of β-catenin, cyclin D1, and c-Myc through the Wnt/β-catenin signaling pathway in the colon. The NF-κB signaling pathway is a pivot of inflammation, while Wnt/β-catenin signaling is essential for tissue development and regeneration. Therefore, inhibiting both paths is crucial for regulating the CRC development process.

It should be noted that there are some limitations to our study, such as the lack of identification of particular species of DRB, since we obtained the DRB from a company that produced bran from different rice varieties. Further studies on the species of DRB and comparisons of different species, such as pigmented and non-pigmented rice varieties, should be performed to confirm the chemopreventive effects. However, further studies on signaling pathways involved in chronic inflammation-induced CRC, such as MAPK, IL-6/STAT3, COX-2/PGE2, and IL-23/Th17, are necessary to investigate the protective effect of DRB in the pathogenesis of colitis-associated CRC. In addition, other cell proliferation markers, such as proliferating cell nuclear antigen (PCNA) and Ki-67, should also be investigated to confirm the protective effects of DRB in the anti-proliferation of cancer cells. Furthermore, DRB contains high levels of dietary fiber, which may be linked with gut microbials, so further studies on the effects of DRB in colitis-associated CRC models linked with gut microbiomes and short-chain fatty acid production should be performed.

## 5. Conclusions

DRB contains several bioactive compounds, such as proteins, vitamins, minerals, dietary fiber, phytic acid, and phenolic acids (especially ferulic acid and *p*-coumaric acid). It exhibited chemopreventive effects in terms of anti-chronic inflammation, anti-cell proliferation, and anti-tumorigenesis in rats with AOM/DSS-induced colitis-associated CRC. DRB supplementation exerted anti-inflammatory activities by reducing DAI scores, pro-inflammatory marker expression (IL-6, NF-κB, and COX-2), and inflammatory cell infiltration in the colon. In addition, DRB supplementation manifested anti-cell proliferation effects by reducing the expression of nuclear β-catenin, cyclin D1, and c-Myc in tumors in colon tissue. Additionally, DRB also suppressed the growth of colon tumorigenesis by delaying preneoplastic ACF and colon tumor development. The chemoprotective effects of DRB on AOM/DSS-induced colitis-associated CRC with respect to chronic inflammation, cell proliferation, and colon tumorigenesis might be attributed to the synergistic actions of the bioactive compounds in DRB. Therefore, these results suggest that DRB could be used as a chemopreventive agent to delay CRC development in rats with colitis-associated CRC. However, the chemopreventive effects of DRB in other species and other mechanistic pathways involved in colitis-associated CRC should be further investigated.

## Figures and Tables

**Table 1:** Criteria of disease activity index (DAI) score.

Score	Weight Loss (%)	Stool Consistency	Rectal Bleeding
0	None	Normal	No bleeding
1	1–5		
2	6–10	Loose	Slight bleeding
3	11–15		
4	>15	Diarrhea	Gross bleeding

**Table 2:** Proximate composition of DRB per 100 g DW (means ± SDs; *n* = 3).

Proximate Composition	Mean ± SD
Energy (kcal)	348.83 ± 3.88
Moisture (g)	5.82 ± 0.27
Protein (g)	16.06 ± 0.47
Total carbohydrates (g)	66.12 ± 0.68
Total fat (g)	2.23 ± 0.49
Ash (g)	9.76 ± 0.21

**Table 3:** Vitamin and mineral contents of DRB per 100 g DW (means ± SDs; *n* = 3).

Micronutrients (mg)	Mean ± SD
Thiamine	2.63 ± 0.21
Niacin	30.31 ± 7.20
Folate	150.00 ± 11.14
Zinc	6.01 ± 0.38
Iron	11.60 ± 1.65
Calcium	46.03 ± 1.28
Potassium	1574.61 ± 20.61
Phosphorus	2176.99 ± 15.33
Magnesium	2217.56 ± 333.22

**Table 4:** Dietary fiber contents of DRB as g/100 g DW (means ± SDs; *n* = 3).

Dietary Fiber Contents (g)	Mean ± SD
TDF	24.00 ± 0.19
IDF	20.68 ± 0.26
SDF	3.32 ± 0.07

**Table 5:** Total phenolic content and phenolic acid profiles of DRB expressed as mg/100 g DW (means ± SDs; *n* = 3).

Phenolic Acid Profiles (mg)	Mean ± SD
Ferulic acid	199.57 ± 5.40
*p*-Coumaric acid	61.61 ± 3.54
Sinapic acid	2.54 ± 0.48
Syringic acid	1.04 ± 0.06
Vanillic acid	1.03 ± 0.17
Caffeic acid	Not detected
4-Hydroxybenzoic acid	Not detected
TPC-HPLC ^1^	265.88 ± 3.11
TPC-FTC ^2^	477.55 ± 18.98

^1^ TPC-HPLC is the sum of all seven individual phenolic acids. ^2^ TPC is the total phenolic content (mg GAE/100 g DRB) determined using the Folin–Ciocalteu assay.

## Data Availability

The datasets generated for this study are available on request from the corresponding author.

## Supplementary Materials

The following supporting information can be downloaded at: https://www.mdpi.com/article/10.3390/foods11213488/s1, Supplementary S1: NF-kB, COX-2 protein band (Western blot Analysis) and Supplementary S2: Phenolic acid chromatograms (HPLC Analysis).
