# Supplementary material for: Inhibitory Effect of Dietary Defatted Rice Bran in an AOM/DSS-Induced Colitis-Associated Colorectal Cancer Experimental Animal Model
Source: Foods. 2022 Nov 2;11(21):3488. doi: 10.3390/foods11213488 (PMC9654186; doi:10.3390/foods11213488)

COX-2

1 2 3 4 5 6

COX-2

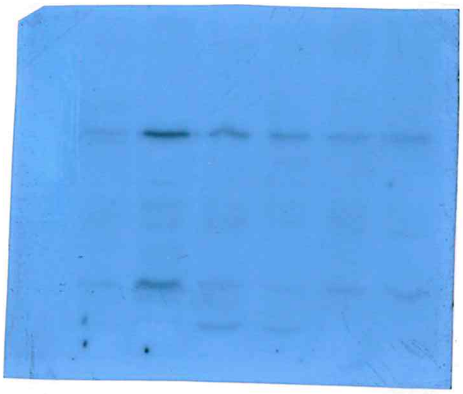

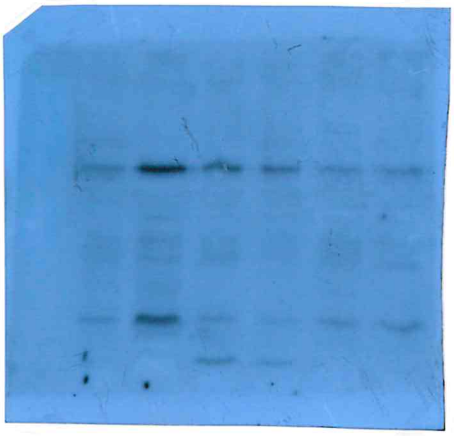

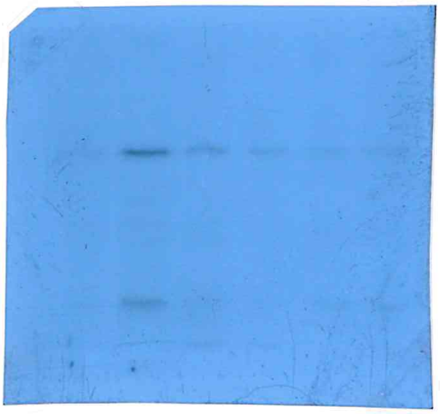

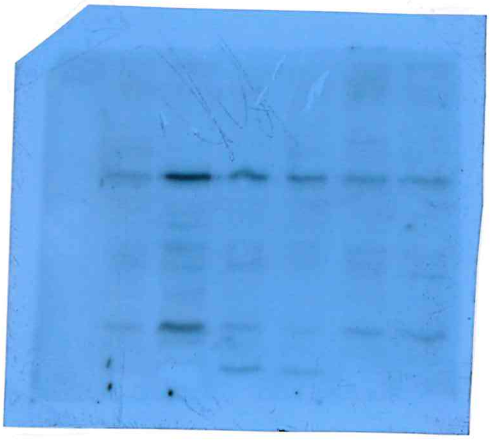

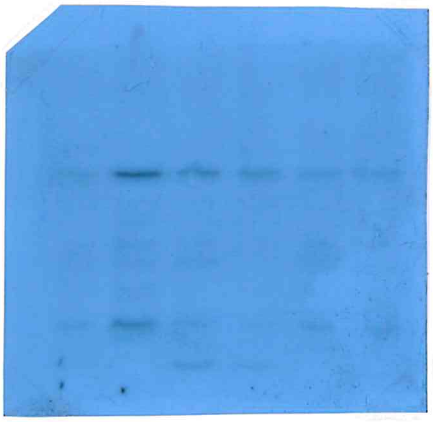

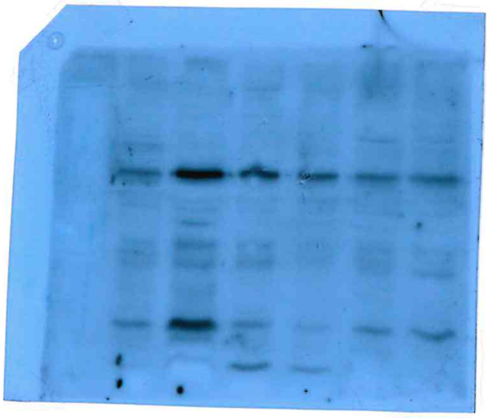

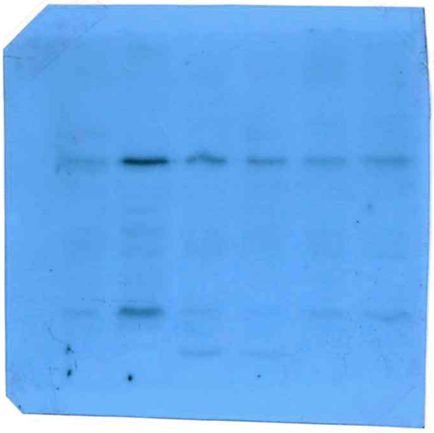

lane 1 Control group
lane 2 AOM/DSS group
lane 3 AOM/DSS + DRB 3 g group
lane 4 AOM/DSS + DRB 6 g group
lane 5 DRB 3 g
lane 6 DRB 6 g

NFKB

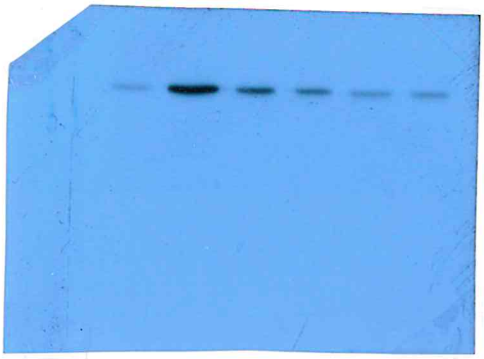

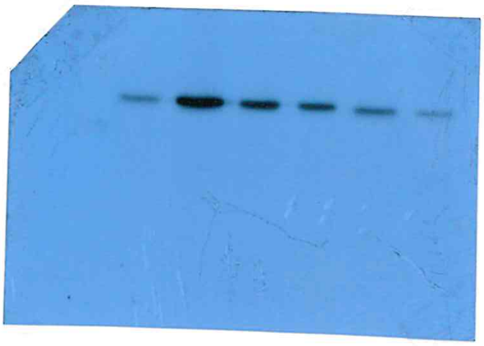

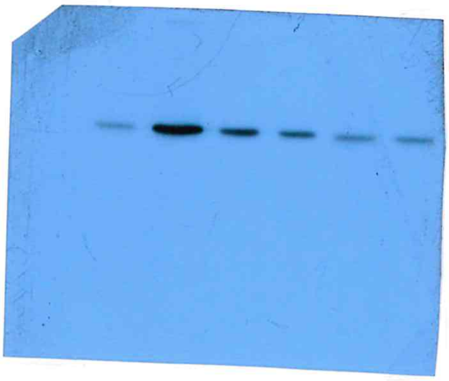

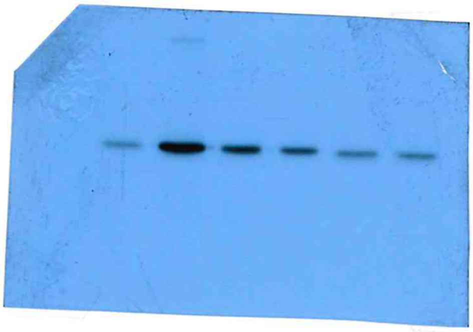

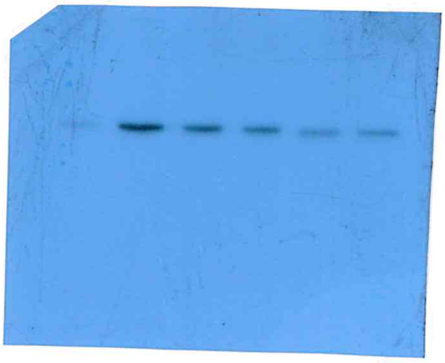

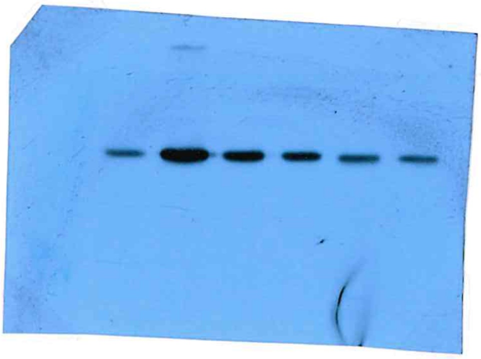

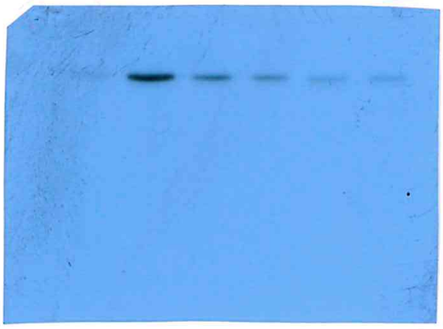

lane 1 Control group
lane 2 AOM/DSS group
lane 3 AOM/DSS + DRB 3 g group
lane 4 AOM/DSS + DRB 6 g group
lane 5 DRB 3 g
lane 6 DRB 6 g

β-actin ของ NFkB 17/2/21

NF-kB

B-actin

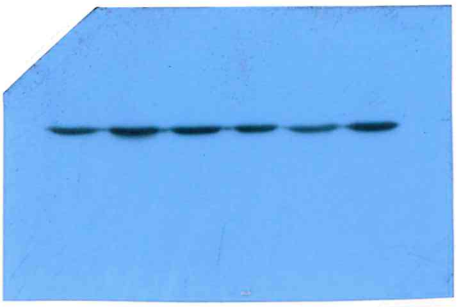

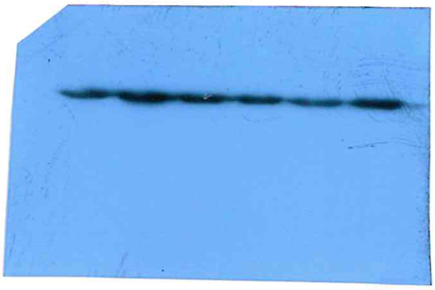

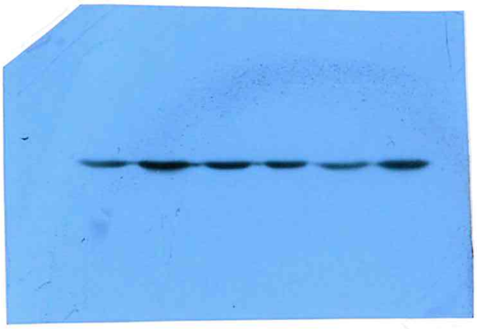

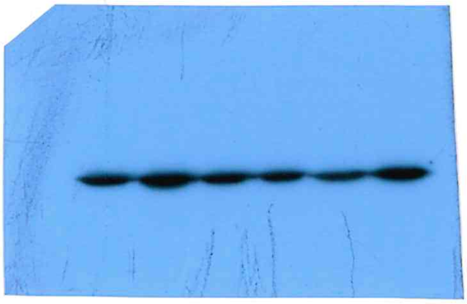

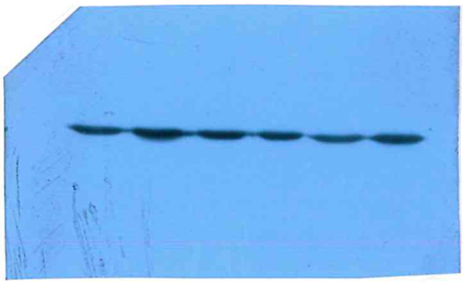

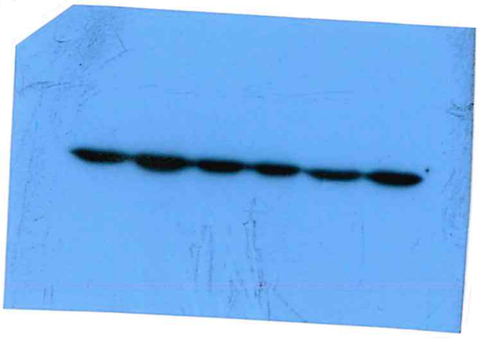

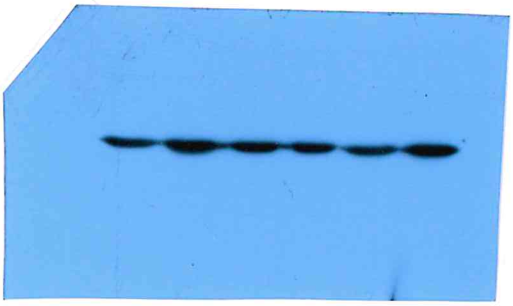

β- actin 409 COX-2 97/12/27

Cox-2

B-actin

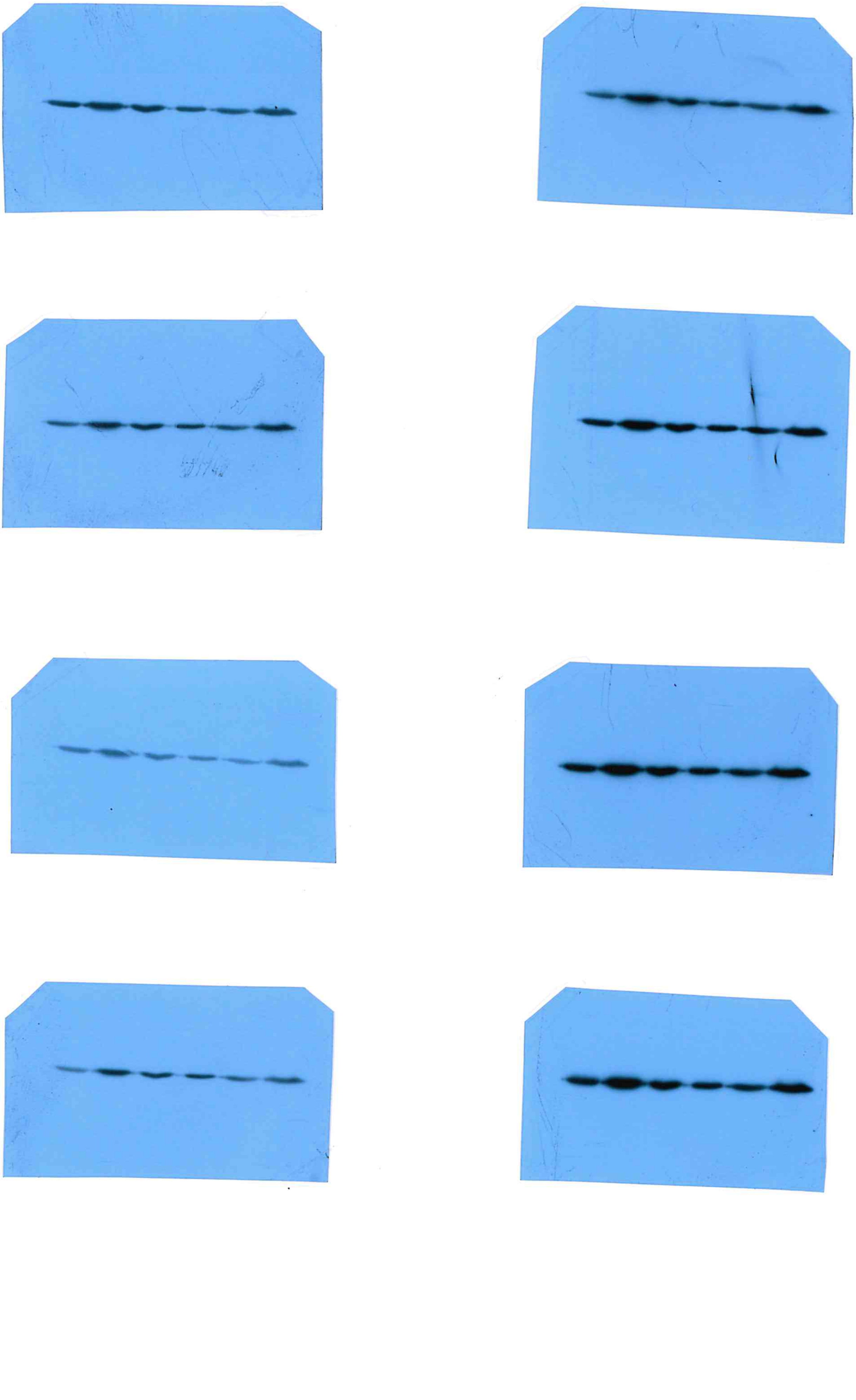

Supplement: Supplementary file 1 [file foods-11-03488-s001.zip › Supplementary S1 NF-kB, COX-2 protein band (Western blot Analysis).pdf]
