# Supplementary material for: Inhibitory Effect of Dietary Defatted Rice Bran in an AOM/DSS-Induced Colitis-Associated Colorectal Cancer Experimental Animal Model
Source: Foods. 2022 Nov 2;11(21):3488. doi: 10.3390/foods11213488 (PMC9654186; doi:10.3390/foods11213488)
Supplement: Supplementary file 1 [file foods-11-03488-s001.zip › Supplementary S2 Phenolic acid chromatograms (HPLC Analysis).pdf]

Phenolic acid Standards

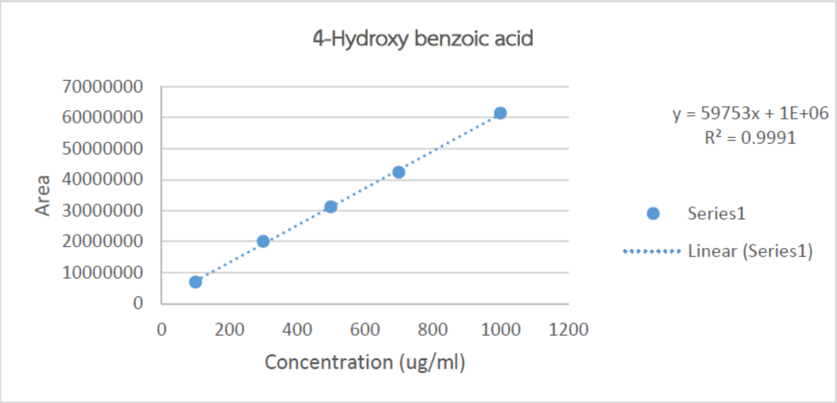

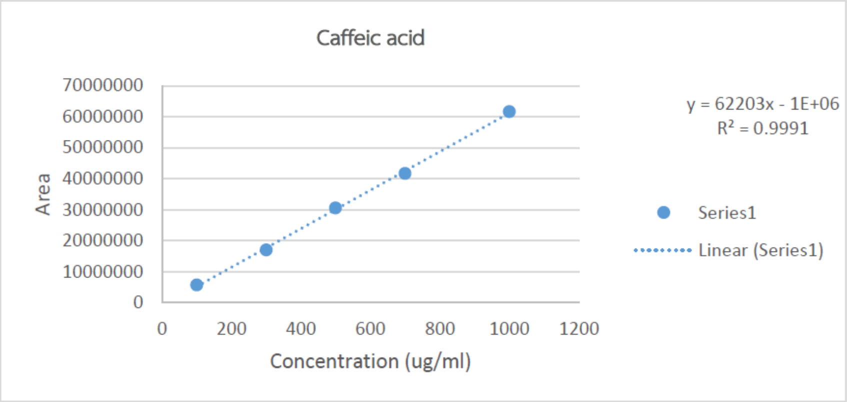

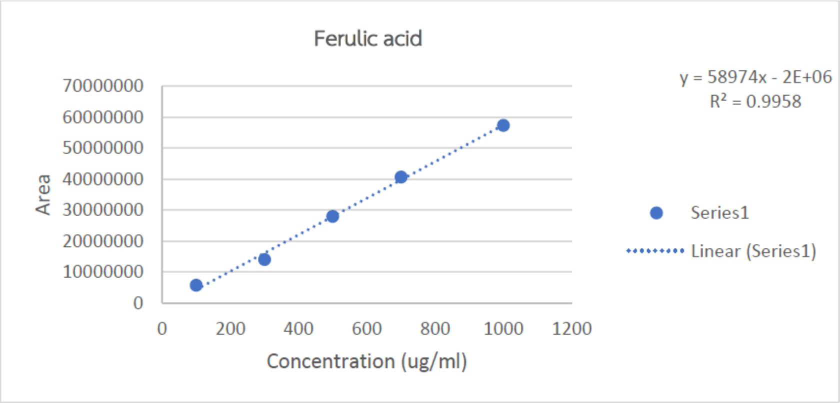

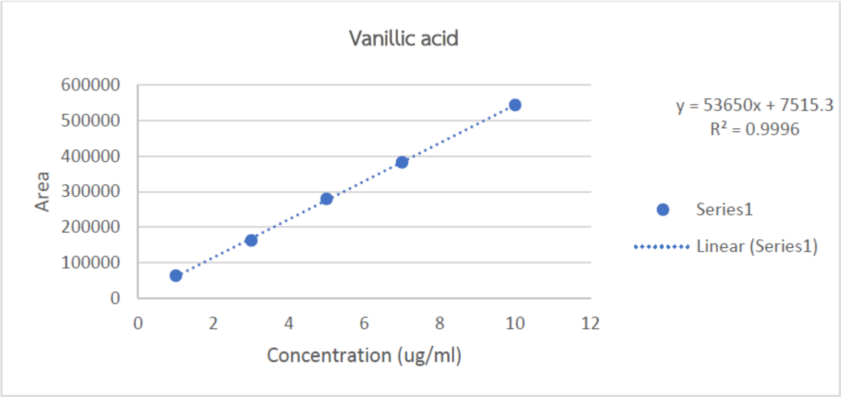

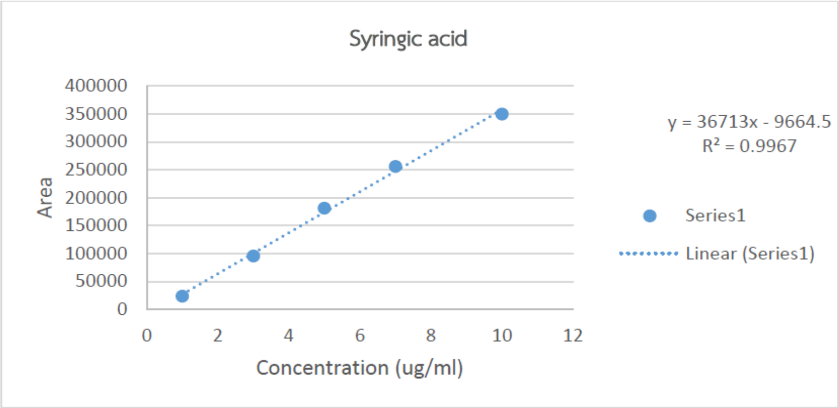

Syringic acid
y = 36713x - 9664.5
R² = 0.9967
Series1
Linear (Series1)
Area
400000
350000
300000
250000
200000
150000
100000
50000
0
0
2
4
6
8
10
12
Concentration (ug/ml)

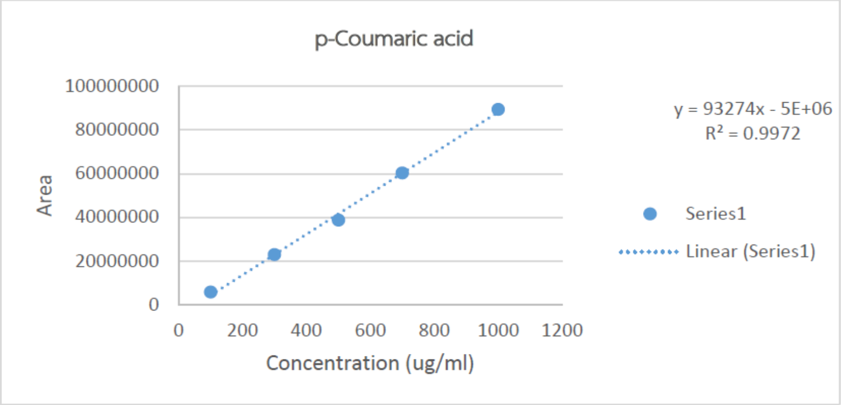

p-Coumaric acid
y = 93274x - 5E+06
R² = 0.9972
Series1
Linear (Series1)
Area
100000000
80000000
60000000
40000000
20000000
0
0
200
400
600
800
1000
1200
Concentration (ug/ml)

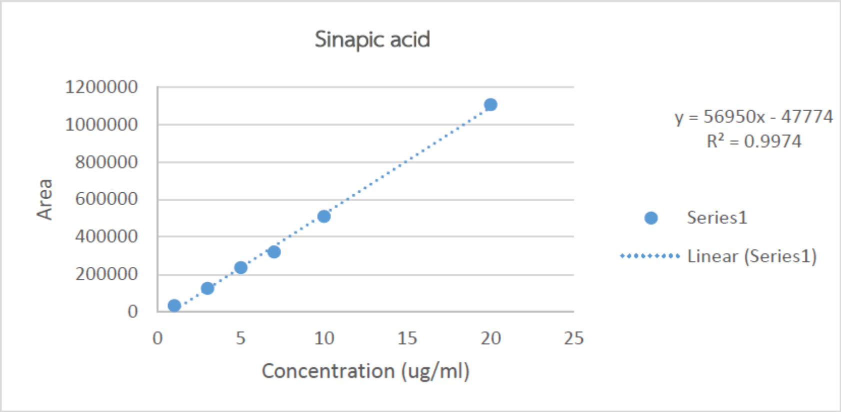

Sinapic acid
y = 56950x - 47774
R² = 0.9974
Series1
Linear (Series1)
Area
1200000
1000000
800000
600000
400000
200000
0
0
5
10
15
20
25
Concentration (ug/ml)

STD	Ret.Time
4-Hydroxybenzolic acid	12.982
Vanillic acid	15.053
p-Courmaric acid	16.996
Ferulic acid	19.056, 21.374
Syringic acid	24.938
Sinapic acid	32.447
Caffeic acid	43.482

ดัดแปลงสภาวะการวิเคราะห์จาก (Sombutsuwan และคณะ, 2021)

เอกสารอ้างอิง

Sombutsuwan, P., Jirattisakul, A., Nakornsadet, A., Akepratumchai, S., Chumsantea, S., Pojjanapornpun, S., Lilitchan, S., Krisnangkura, K. and Aryusuk, K., 2021, "A Simple and Efficient Method for Synthesis and Extraction of Ethyl Ferulate from γ-Oryzanol", **Journal of Oleo Science**, Vol. 70, No. 6, pp. 757-767.

==== Shimadzu LCsolution Analysis Report ====

E:\Pang\phenolic acid\2021-12-03 analyze sample\sample 1.1 3-12-2021.lcd

Acquired by	: Admin
Sample Name	: sample 1.1 3-12-2021 Mightysil 70Hex30EtAc0.2aa
Sample ID	: sample 1.1 3-12-21
Tray#	: 1
Vail #	: 1
Injection Volume	: 10 uL
Data File Name	: sample 1.1 3-12-2021.lcd
Method File Name	: phenolic acid 275.lcm
Batch File Name	:
Report File Name	: ethyl ferulate wavelength 275.lcr
Data Acquired	: 3/12/2564 9:57:49
Data Processed	: 21/12/2564 10:49:32

<Chromatogram>

sample 1.1 3-12-2021 Mightysil 70Hex30EtAc0.2aa

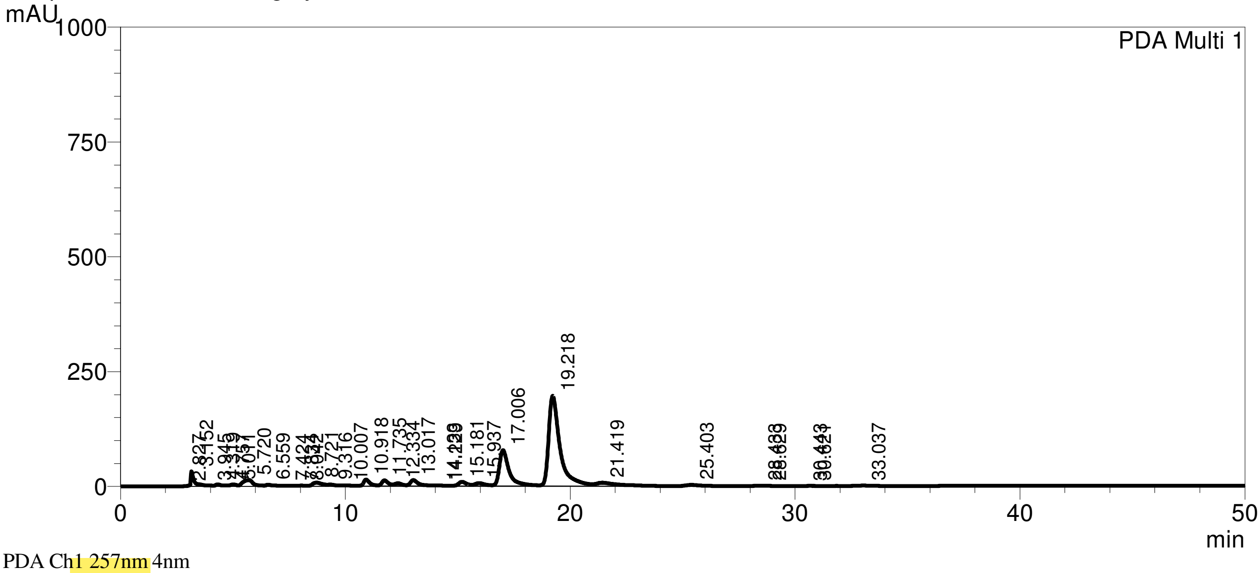

PDA Ch1 257nm 4nm

Peak#	Ret. Time	Area	Height	Area %	Height %
1	2.827	4023	405	0.032	0.097
2	3.152	415192	32811	3.288	7.898
3	3.945	3312	377	0.026	0.091
4	4.319	80409	4244	0.637	1.021
5	4.757	15468	1943	0.122	0.468
6	5.011	78094	4142	0.618	0.997
7	5.720	434121	13305	3.438	3.202
8	6.559	81992	2995	0.649	0.721
9	7.424	8060	537	0.064	0.129
10	7.834	17529	946	0.139	0.228
11	8.042	12214	1254	0.097	0.302
12	8.721	231424	7343	1.833	1.767
13	9.316	70738	3114	0.560	0.749
14	10.007	36319	1435	0.288	0.345
15	10.918	266549	13751	2.111	3.310
16	11.735	236966	11830	1.877	2.848
17	12.334	125713	5562	0.996	1.339
18	13.017	300632	12329	2.381	2.968
19	14.133	4439	379	0.035	0.091
20	14.229	3611	312	0.029	0.075
21	15.181	196982	8338	1.560	2.007
22	15.937	171365	5735	1.357	1.381
23	17.006	2317488	77321	18.354	18.611
24	19.218	7157891	195808	56.687	47.131
25	21.419	152197	3883	1.205	0.935
26	25.403	121233	2579	0.960	0.621
27	28.433	18709	706	0.148	0.170
28	28.629	16388	625	0.130	0.151
29	30.443	2914	261	0.023	0.063
30	30.621	8005	340	0.063	0.082

Peak#	Ret. Time	Area	Height	Area %	Height %
31	33.037	36959	844	0.293	0.203
Total		12626931	415453	100.000	100.000

==== Shimadzu LCsolution Analysis Report ====

E:\Pang\phenolic acid\2021-12-03 analyze sample\sample 1.1 3-12-2021.lcd

Acquired by	: Admin
Sample Name	: sample 1.1 3-12-2021 Mightysil 70Hex30EtAc0.2aa
Sample ID	: sample 1.1 3-12-21
Tray#	: 1
Vail #	: 1
Injection Volume	: 10 uL
Data File Name	: sample 1.1 3-12-2021.lcd
Method File Name	: phenolic acid 275.lcm
Batch File Name	:
Report File Name	: ethyl ferulate wavelength 275.lcr
Data Acquired	: 3/12/2564 9:57:49
Data Processed	: 21/12/2564 10:49:32

<Chromatogram>

sample 1.1 3-12-2021 Mightysil 70Hex30EtAc0.2aa

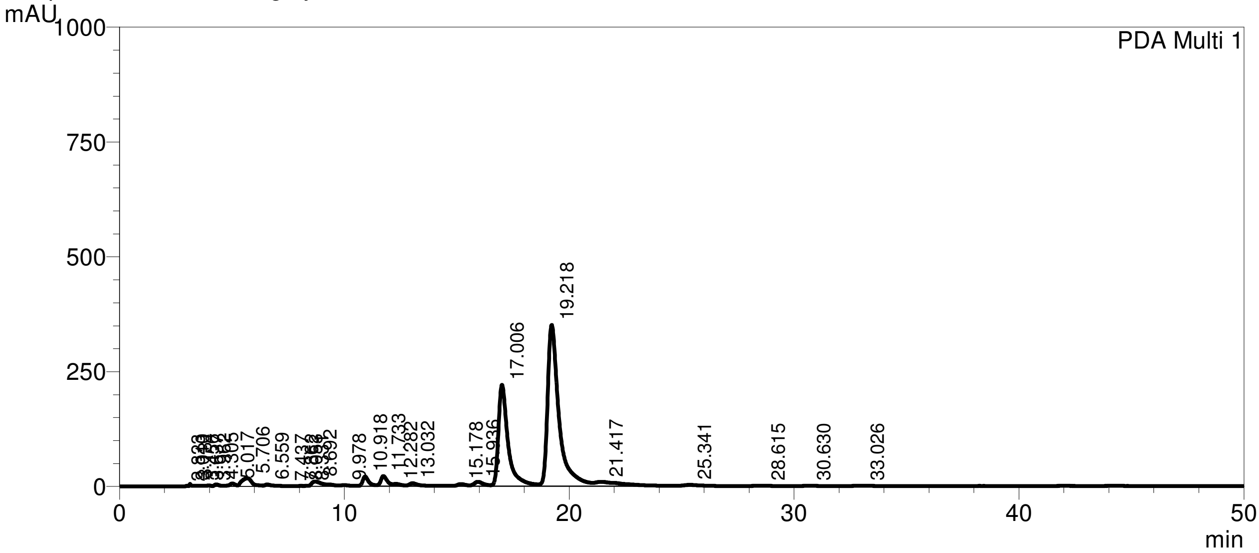

PDA Ch1 271nm 4nm

Peak#	Ret. Time	Area	Height	Area %	Height %
1	2.823	3741	401	0.016	0.057
2	3.010	11001	1512	0.048	0.215
3	3.131	27489	5532	0.121	0.788
4	3.456	27345	1686	0.120	0.240
5	3.637	27908	1727	0.123	0.246
6	3.962	25583	2032	0.113	0.289
7	4.305	69989	4428	0.308	0.630
8	5.017	118636	6271	0.523	0.893
9	5.706	559624	17399	2.466	2.477
10	6.559	90841	3844	0.400	0.547
11	7.437	9736	519	0.043	0.074
12	7.862	4323	336	0.019	0.048
13	8.053	6560	677	0.029	0.096
14	8.331	6208	571	0.027	0.081
15	8.692	376319	9974	1.658	1.420
16	9.978	47372	2043	0.209	0.291
17	10.918	380138	20383	1.675	2.902
18	11.733	431162	21444	1.900	3.053
19	12.282	93499	4182	0.412	0.595
20	13.032	137444	5586	0.606	0.795
21	15.178	92299	4004	0.407	0.570
22	15.936	249425	8917	1.099	1.270
23	17.006	6542344	220018	28.825	31.325
24	19.218	12926605	350623	56.953	49.920
25	21.417	179585	3275	0.791	0.466
26	25.341	115742	2251	0.510	0.321
27	28.615	52479	896	0.231	0.128
28	30.630	31971	783	0.141	0.111
29	33.026	51627	1049	0.227	0.149
Total		22696994	702364	100.000	100.000

==== Shimadzu LCsolution Analysis Report ====

E:\Pang\phenolic acid\2021-12-03 analyze sample\sample 1.1 3-12-2021.lcd

Acquired by	: Admin
Sample Name	: sample 1.1 3-12-2021 Mightysil 70Hex30EtAc0.2aa
Sample ID	: sample 1.1 3-12-21
Tray#	: 1
Vail #	: 1
Injection Volume	: 10 uL
Data File Name	: sample 1.1 3-12-2021.lcd
Method File Name	: phenolic acid 275.lcm
Batch File Name	:
Report File Name	: ethyl ferulate wavelength 275.lcr
Data Acquired	: 3/12/2564 9:57:49
Data Processed	: 21/12/2564 10:49:32

<Chromatogram>

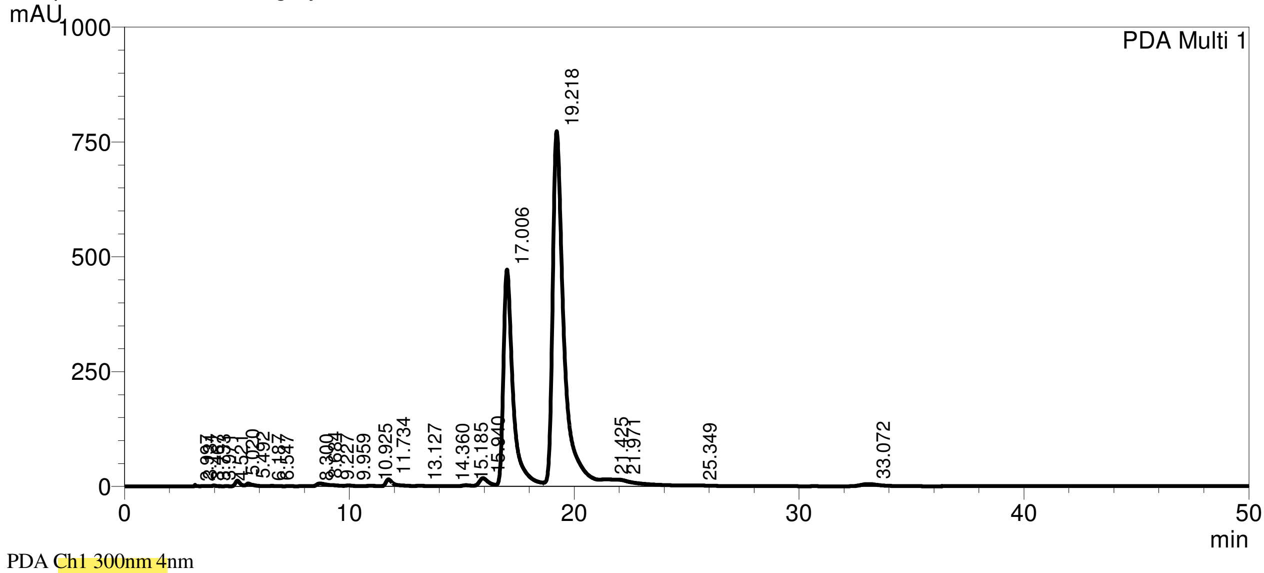

PDA Ch1 300nm 4nm

Peak#	Ret. Time	Area	Height	Area %	Height %
1	2.997	1210	118	0.003	0.009
2	3.131	14160	3563	0.032	0.269
3	3.467	6547	374	0.015	0.028
4	3.733	3080	323	0.007	0.024
5	3.971	17389	1709	0.039	0.129
6	4.521	1871	173	0.004	0.013
7	5.020	177841	11953	0.400	0.901
8	5.492	122599	6046	0.275	0.456
9	6.187	9615	657	0.022	0.050
10	6.547	14812	781	0.033	0.059
11	8.300	6637	395	0.015	0.030
12	8.684	172929	5728	0.389	0.432
13	9.227	39137	2192	0.088	0.165
14	9.959	25981	1442	0.058	0.109
15	10.925	14251	775	0.032	0.058
16	11.734	326839	14650	0.734	1.104
17	13.127	16188	679	0.036	0.051
18	14.360	6477	328	0.015	0.025
19	15.185	41989	1881	0.094	0.142
20	15.940	481053	17133	1.081	1.292
21	17.006	13997071	471647	31.449	35.559
22	19.218	28526357	772346	64.094	58.230
23	21.425	82698	2412	0.186	0.182
24	21.971	105217	3557	0.236	0.268
25	25.349	31135	764	0.070	0.058
26	33.072	263660	4746	0.592	0.358
Total		44506743	1326369	100.000	100.000

==== Shimadzu LCsolution Analysis Report ====

E:\Pang\phenolic acid\2021-12-03 analyze sample\sample 1.1 3-12-2021.lcd

Acquired by	: Admin
Sample Name	: sample 1.1 3-12-2021 Mightysil 70Hex30EtAc0.2aa
Sample ID	: sample 1.1 3-12-21
Tray#	: 1
Vail #	: 1
Injection Volume	: 10 uL
Data File Name	: sample 1.1 3-12-2021.lcd
Method File Name	: phenolic acid 275.lcm
Batch File Name	:
Report File Name	: ethyl ferulate wavelength 275.lcr
Data Acquired	: 3/12/2564 9:57:49
Data Processed	: 21/12/2564 10:49:32

<Chromatogram>

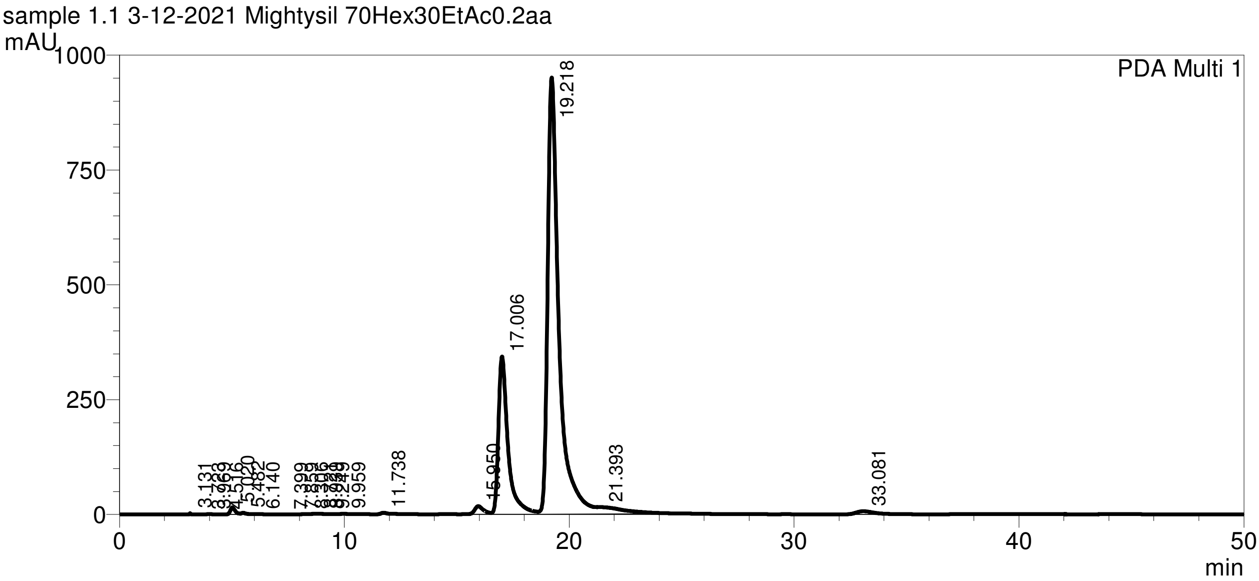

PDA Ch1 320nm 4nm

Peak#	Ret. Time	Area	Height	Area %	Height %
1	3.131	9458	2998	0.021	0.222
2	3.723	1055	113	0.002	0.008
3	3.969	11242	1013	0.024	0.075
4	4.516	2446	216	0.005	0.016
5	5.020	229044	14950	0.499	1.107
6	5.482	52250	3390	0.114	0.251
7	6.140	10765	512	0.023	0.038
8	7.399	2395	171	0.005	0.013
9	7.859	1483	132	0.003	0.010
10	8.306	6653	416	0.014	0.031
11	8.721	22819	1073	0.050	0.079
12	8.939	12358	957	0.027	0.071
13	9.249	13834	698	0.030	0.052
14	9.959	22217	1398	0.048	0.104
15	11.738	86016	3244	0.187	0.240
16	15.950	482489	17353	1.050	1.285
17	17.006	10200369	343232	22.202	25.425
18	19.218	34302873	950418	74.662	70.403
19	21.393	84045	938	0.183	0.070
20	33.081	390186	6742	0.849	0.499
Total		45943995	1349963	100.000	100.000

==== Shimadzu LCsolution Analysis Report ====

E:\Pang\phenolic acid\2021-12-03 analyze sample\sample 1.2_3-12-2021.lcd

Acquired by	: Admin
Sample Name	: sample 1.2_3-12-2021_Mightysil_70Hex-30EtAc-0.2aa
Sample ID	: sample 1.2_3-12-21
Tray#	: 1
Vail #	: 1
Injection Volume	: 10 uL
Data File Name	: sample 1.2_3-12-2021.lcd
Method File Name	: phenolic acid 275.lcm
Batch File Name	:
Report File Name	: ethyl ferulate wavelength 275.lcr
Data Acquired	: 3/12/2564 11:39:23
Data Processed	: 21/12/2564 10:51:57

<Chromatogram>

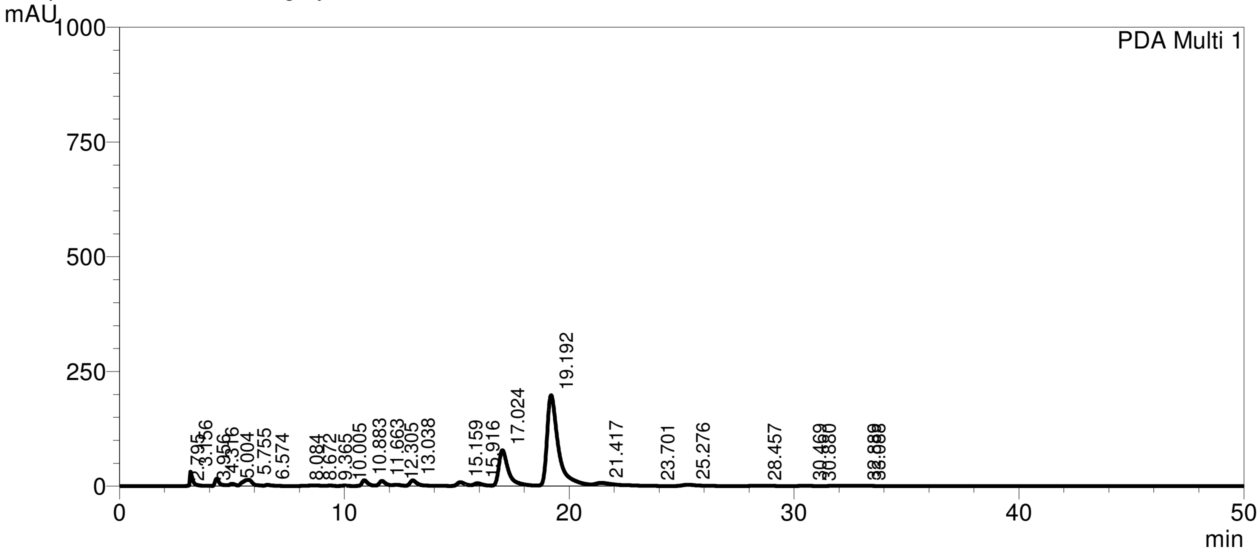

1 PDA Multi 1/257nm 4nm

PDA Ch1 257nm 4nm

Peak#	Ret. Time	Area	Height	Area %	Height %
1	2.795	2697	251	0.022	0.061
2	3.156	341550	31920	2.735	7.717
3	3.956	1889	200	0.015	0.048
4	4.316	240859	16430	1.929	3.972
5	5.004	106495	5405	0.853	1.307
6	5.755	438598	13725	3.512	3.318
7	6.574	58703	2660	0.470	0.643
8	8.084	5115	639	0.041	0.155
9	8.672	50963	1315	0.408	0.318
10	9.365	22314	1104	0.179	0.267
11	10.005	21168	1040	0.170	0.251
12	10.883	237325	12731	1.900	3.078
13	11.663	231570	11134	1.854	2.692
14	12.305	72169	2950	0.578	0.713
15	13.038	305940	12138	2.450	2.935
16	15.159	207222	8362	1.659	2.022
17	15.916	172623	5968	1.382	1.443
18	17.024	2340767	78167	18.744	18.899
19	19.192	7246447	198106	58.026	47.897
20	21.417	161675	3963	1.295	0.958
21	23.701	2599	-7	0.021	-0.002
22	25.276	134818	2832	1.080	0.685
23	28.457	47594	908	0.381	0.220
24	30.469	12420	380	0.099	0.092
25	30.880	1196	162	0.010	0.039
26	32.889	14836	593	0.119	0.143
27	33.088	8701	533	0.070	0.129
Total		12488253	413611	100.000	100.000

==== Shimadzu LCsolution Analysis Report ====

E:\Pang\phenolic acid\2021-12-03 analyze sample\sample 1.2_3-12-2021.lcd

Acquired by	: Admin
Sample Name	: sample 1.2_3-12-2021_Mightysil_70Hex-30EtAc-0.2aa
Sample ID	: sample 1.2_3-12-21
Tray#	: 1
Vail #	: 1
Injection Volume	: 10 uL
Data File Name	: sample 1.2_3-12-2021.lcd
Method File Name	: phenolic acid 275.lcm
Batch File Name	:
Report File Name	: ethyl ferulate wavelength 275.lcr
Data Acquired	: 3/12/2564 11:39:23
Data Processed	: 21/12/2564 10:51:57

<Chromatogram>

sample 1.2_3-12-2021_Mightysil_70Hex-30EtAc-0.2aa

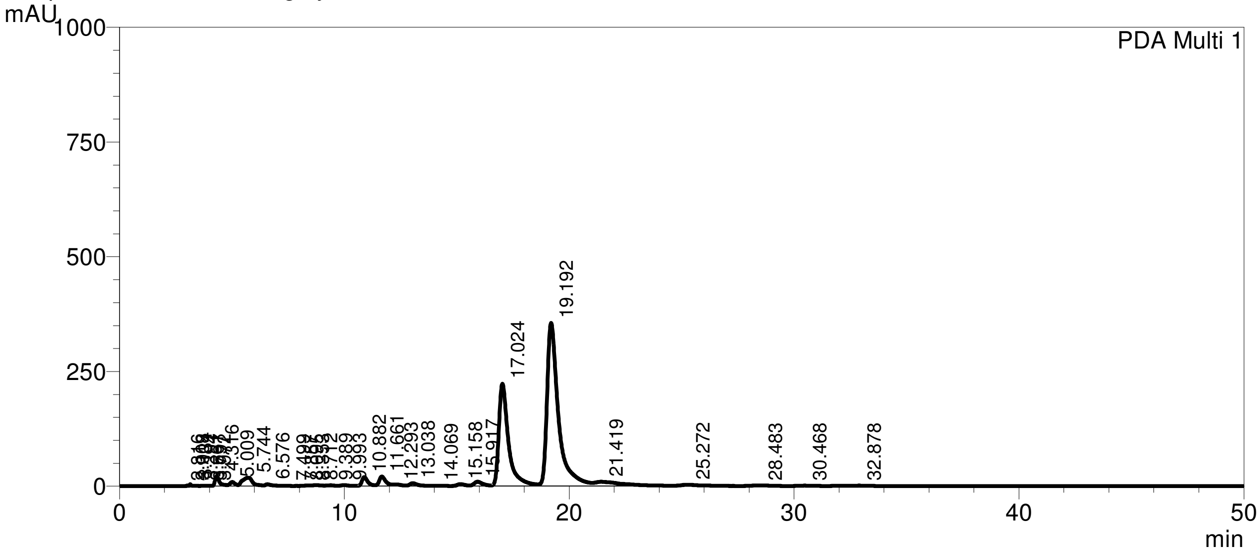

1 PDA Multi 1/271nm 4nm

PDA Ch1 271nm 4nm

Peak#	Ret. Time	Area	Height	Area %	Height %
1	2.816	2945	300	0.013	0.042
2	3.008	9970	1387	0.044	0.193
3	3.134	23464	4602	0.102	0.640
4	3.394	12636	980	0.055	0.136
5	3.661	8369	824	0.037	0.115
6	3.797	4325	772	0.019	0.107
7	3.972	14543	1048	0.064	0.146
8	4.316	317133	21714	1.385	3.021
9	5.009	153760	8887	0.671	1.236
10	5.744	594444	18316	2.596	2.548
11	6.576	95066	3958	0.415	0.551
12	7.499	8903	482	0.039	0.067
13	7.857	4385	367	0.019	0.051
14	8.095	8543	932	0.037	0.130
15	8.353	19412	1211	0.085	0.169
16	8.712	53686	1937	0.234	0.270
17	9.389	39098	1623	0.171	0.226
18	9.993	41881	1885	0.183	0.262
19	10.882	355500	19233	1.553	2.676
20	11.661	423626	20433	1.850	2.843
21	12.293	79135	3336	0.346	0.464
22	13.038	135689	5810	0.593	0.808
23	14.069	1044	93	0.005	0.013
24	15.158	92081	3953	0.402	0.550
25	15.917	246796	9138	1.078	1.271
26	17.024	6642249	222700	29.008	30.982
27	19.192	13132682	354922	57.353	49.377
28	21.419	143677	3155	0.627	0.439

Peak#	Ret. Time	Area	Height	Area %	Height %
29	25.272	115327	2294	0.504	0.319
30	28.483	64071	1161	0.280	0.162
31	30.468	31256	773	0.136	0.108
32	32.878	22407	578	0.098	0.080
Total		22898105	718803	100.000	100.000

==== Shimadzu LCsolution Analysis Report ====

E:\Pang\phenolic acid\2021-12-03 analyze sample\sample 1.2_3-12-2021.lcd

Acquired by	: Admin
Sample Name	: sample 1.2_3-12-2021_Mightysil_70Hex-30EtAc-0.2aa
Sample ID	: sample 1.2_3-12-21
Tray#	: 1
Vail #	: 1
Injection Volume	: 10 uL
Data File Name	: sample 1.2_3-12-2021.lcd
Method File Name	: phenolic acid 275.lcm
Batch File Name	:
Report File Name	: ethyl ferulate wavelength 275.lcr
Data Acquired	: 3/12/2564 11:39:23
Data Processed	: 21/12/2564 10:51:57

<Chromatogram>

sample 1.2_3-12-2021_Mightysil_70Hex-30EtAc-0.2aa

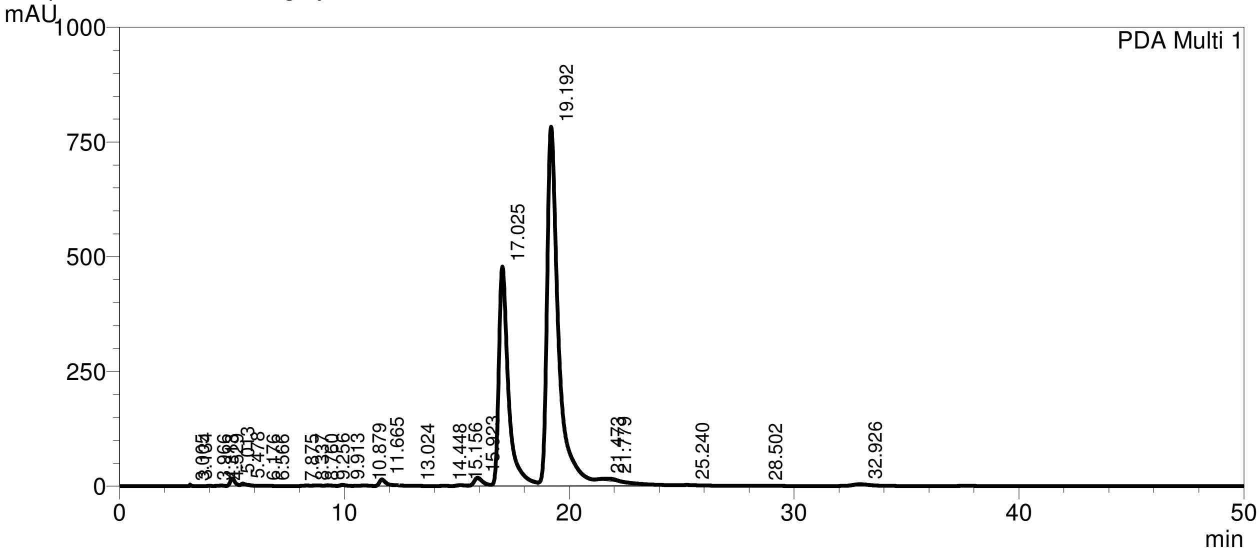

1 PDA Multi 1/300nm 4nm

PDA Ch1 300nm 4nm

Peak#	Ret. Time	Area	Height	Area %	Height %
1	3.005	1169	149	0.003	0.011
2	3.134	16779	3375	0.038	0.251
3	3.966	13313	813	0.030	0.060
4	4.313	5532	568	0.012	0.042
5	4.529	18239	1313	0.041	0.098
6	5.013	258485	16780	0.578	1.247
7	5.478	114866	5470	0.257	0.407
8	6.176	12929	825	0.029	0.061
9	6.566	14607	795	0.033	0.059
10	7.875	3513	250	0.008	0.019
11	8.337	24167	1188	0.054	0.088
12	8.760	37196	1376	0.083	0.102
13	9.256	32350	1633	0.072	0.121
14	9.913	44054	2474	0.098	0.184
15	10.879	36241	1255	0.081	0.093
16	11.665	347494	14188	0.777	1.054
17	13.024	8175	489	0.018	0.036
18	14.448	7392	390	0.017	0.029
19	15.156	43267	1866	0.097	0.139
20	15.923	473824	17642	1.059	1.311
21	17.025	14195548	477643	31.732	35.497
22	19.192	28553398	782250	63.826	58.134
23	21.473	60814	3207	0.136	0.238
24	21.779	172221	4734	0.385	0.352
25	25.240	27934	780	0.062	0.058
26	28.502	20358	486	0.046	0.036
27	32.926	192447	3648	0.430	0.271
Total		44736310	1345588	100.000	100.000

==== Shimadzu LCsolution Analysis Report ====

E:\Pang\phenolic acid\2021-12-03 analyze sample\sample 1.2_3-12-2021.lcd

Acquired by	: Admin
Sample Name	: sample 1.2_3-12-2021_Mightysil_70Hex-30EtAc-0.2aa
Sample ID	: sample 1.2_3-12-21
Tray#	: 1
Vail #	: 1
Injection Volume	: 10 uL
Data File Name	: sample 1.2_3-12-2021.lcd
Method File Name	: phenolic acid 275.lcm
Batch File Name	:
Report File Name	: ethyl ferulate wavelength 275.lcr
Data Acquired	: 3/12/2564 11:39:23
Data Processed	: 21/12/2564 10:51:57

<Chromatogram>

sample 1.2_3-12-2021_Mightysil_70Hex-30EtAc-0.2aa

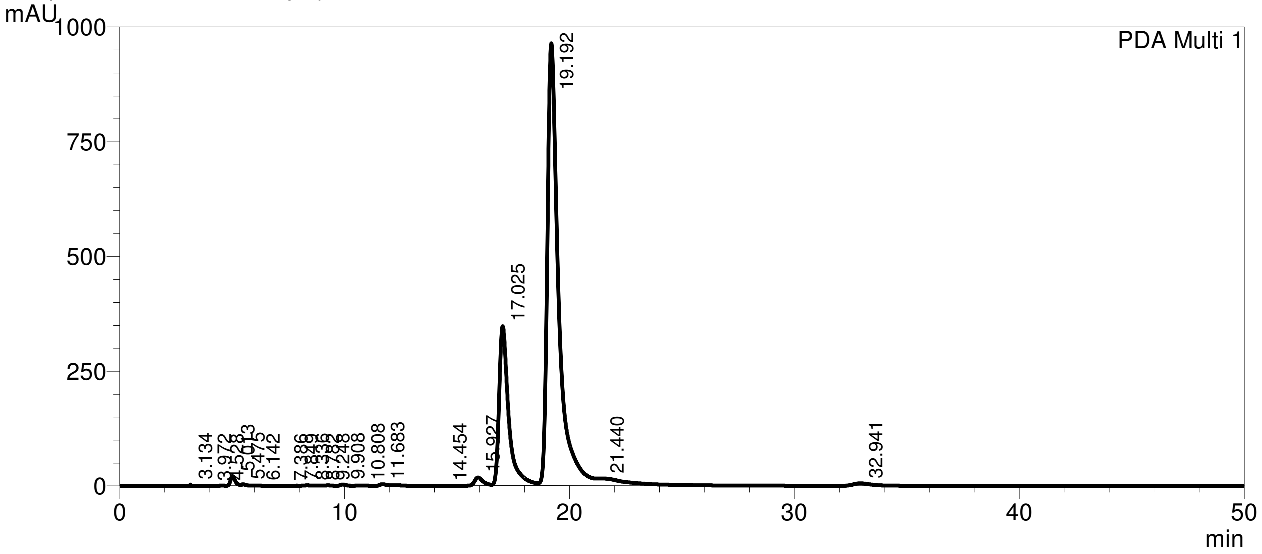

1 PDA Multi 1/320nm 4nm

PDA Ch1 320nm 4nm

Peak#	Ret. Time	Area	Height	Area %	Height %
1	3.134	13825	3060	0.030	0.222
2	3.972	8926	508	0.019	0.037
3	4.528	16226	1100	0.035	0.080
4	5.013	324562	20875	0.694	1.517
5	5.475	54560	3562	0.117	0.259
6	6.142	13072	606	0.028	0.044
7	7.386	3336	216	0.007	0.016
8	7.849	5119	329	0.011	0.024
9	8.336	25371	1168	0.054	0.085
10	8.782	20676	857	0.044	0.062
11	9.248	20409	1114	0.044	0.081
12	9.908	51318	3001	0.110	0.218
13	10.808	21750	600	0.046	0.044
14	11.683	59990	3036	0.128	0.221
15	14.454	4226	217	0.009	0.016
16	15.927	486367	17974	1.040	1.306
17	17.025	10366027	347737	22.157	25.264
18	19.192	34865337	963340	74.522	69.989
19	21.440	100394	1668	0.215	0.121
20	32.941	323856	5456	0.692	0.396
Total		46785347	1376423	100.000	100.000

==== Shimadzu LCsolution Analysis Report ====

E:\Pang\phenolic acid\2021-12-03 analyze sample\sample 2.1_3-12-2021.lcd

Acquired by	: Admin
Sample Name	: sample 2.1_3-12-2021_Mightysil_70Hex-30EtAc-0.2aa
Sample ID	: sample 2.1_3-12-21
Tray#	: 1
Vail #	: 1
Injection Volume	: 10 uL
Data File Name	: sample 2.1_3-12-2021.lcd
Method File Name	: phenolic acid 275.lcm
Batch File Name	:
Report File Name	: ethyl ferulate wavelength 275.lcr
Data Acquired	: 3/12/2564 12:35:48
Data Processed	: 21/12/2564 10:53:28

<Chromatogram>

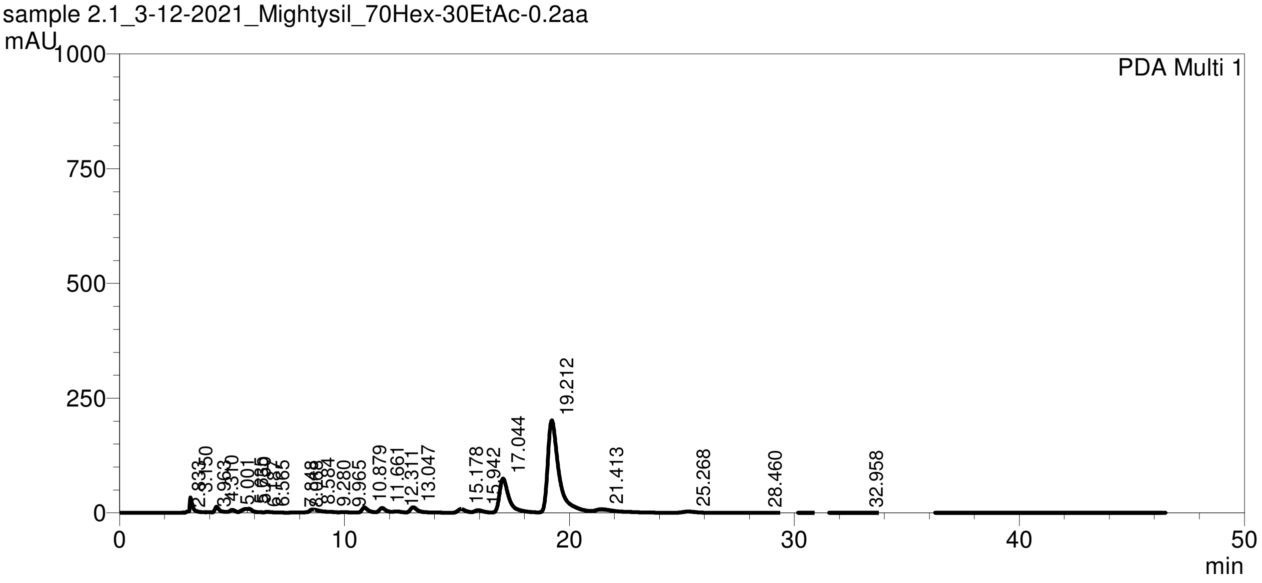

1 PDA Multi 1/257nm 4nm

PDA Ch1 257nm 4nm

Peak#	Ret. Time	Area	Height	Area %	Height %
1	2.833	10811	869	0.087	0.207
2	3.150	397795	33626	3.188	7.998
3	3.963	4640	518	0.037	0.123
4	4.310	213319	13215	1.709	3.143
5	5.001	117223	6437	0.939	1.531
6	5.635	145622	8546	1.167	2.033
7	5.750	131957	8720	1.057	2.074
8	6.187	18498	1517	0.148	0.361
9	6.565	46384	1953	0.372	0.464
10	7.848	4990	347	0.040	0.083
11	8.068	4363	545	0.035	0.130
12	8.584	227877	6920	1.826	1.646
13	9.280	41920	2105	0.336	0.501
14	9.965	31718	1101	0.254	0.262
15	10.879	220244	11351	1.765	2.700
16	11.661	210826	10089	1.689	2.400
17	12.311	68011	2837	0.545	0.675
18	13.047	278389	11752	2.231	2.795
19	15.178	190474	8051	1.526	1.915
20	15.942	152909	5560	1.225	1.322
21	17.044	2206977	74483	17.685	17.715
22	19.212	7373402	201123	59.083	47.836
23	21.413	184676	4433	1.480	1.054
24	25.268	124351	2821	0.996	0.671
25	28.460	44335	871	0.355	0.207
26	32.958	27961	654	0.224	0.156
Total		12479671	420446	100.000	100.000

==== Shimadzu LCsolution Analysis Report ====

E:\Pang\phenolic acid\2021-12-03 analyze sample\sample 2.1_3-12-2021.lcd

Acquired by	: Admin
Sample Name	: sample 2.1_3-12-2021_Mightysil_70Hex-30EtAc-0.2aa
Sample ID	: sample 2.1_3-12-21
Tray#	: 1
Vail #	: 1
Injection Volume	: 10 uL
Data File Name	: sample 2.1_3-12-2021.lcd
Method File Name	: phenolic acid 275.lcm
Batch File Name	:
Report File Name	: ethyl ferulate wavelength 275.lcr
Data Acquired	: 3/12/2564 12:35:48
Data Processed	: 21/12/2564 10:53:28

<Chromatogram>

sample 2.1_3-12-2021_Mightysil_70Hex-30EtAc-0.2aa

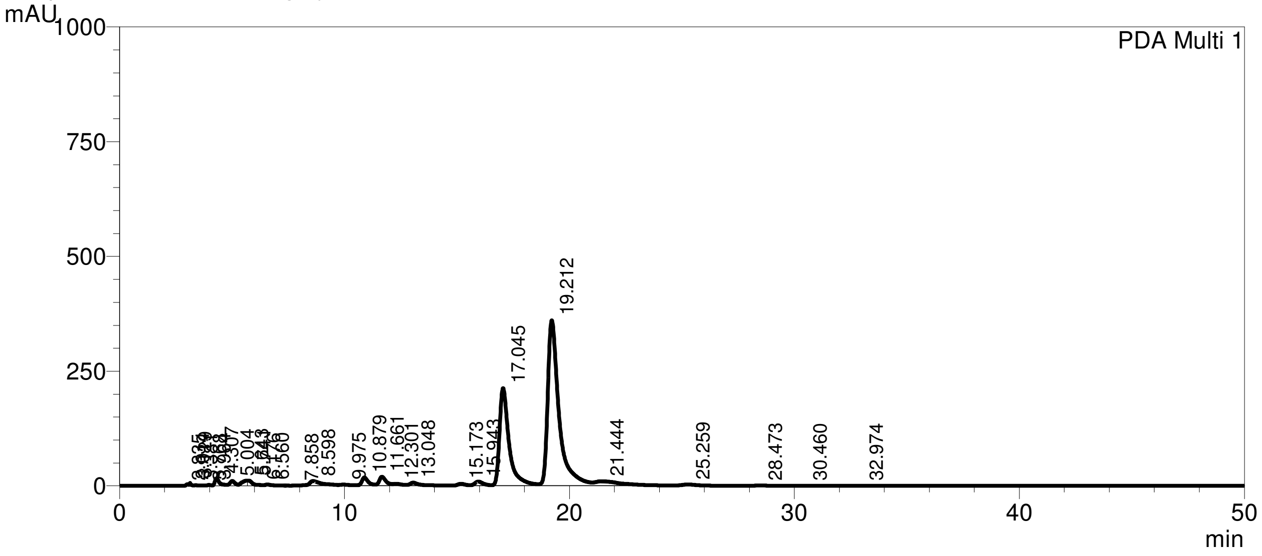

1 PDA Multi 1/271nm 4nm

PDA Ch1 271nm 4nm

Peak#	Ret. Time	Area	Height	Area %	Height %
1	2.835	7325	751	0.032	0.104
2	3.014	29477	3709	0.130	0.514
3	3.129	33778	6684	0.149	0.926
4	3.381	20785	1529	0.092	0.212
5	3.768	17624	1208	0.078	0.167
6	3.964	25933	2044	0.114	0.283
7	4.307	244834	16348	1.081	2.266
8	5.004	176381	10524	0.779	1.459
9	5.643	190176	11114	0.840	1.540
10	5.741	165092	11273	0.729	1.562
11	6.176	22912	1802	0.101	0.250
12	6.560	53910	2543	0.238	0.353
13	7.858	2188	239	0.010	0.033
14	8.598	393095	9825	1.735	1.362
15	9.975	60270	1997	0.266	0.277
16	10.879	346945	17461	1.532	2.420
17	11.661	406834	19032	1.796	2.638
18	12.301	90470	3582	0.399	0.496
19	13.048	159770	6115	0.705	0.847
20	15.173	86719	3807	0.383	0.528
21	15.943	228199	8755	1.007	1.213
22	17.045	6286278	212408	27.751	29.439
23	19.212	13164239	359877	58.114	49.877
24	21.444	213309	3979	0.942	0.551
25	25.259	107084	2346	0.473	0.325
26	28.473	54403	1051	0.240	0.146
27	30.460	37356	876	0.165	0.121

Peak#	Ret. Time	Area	Height	Area %	Height %
28	32.974	27116	646	0.120	0.090
Total		22652503	721525	100.000	100.000

==== Shimadzu LCsolution Analysis Report ====

E:\Pang\phenolic acid\2021-12-03 analyze sample\sample 2.1_3-12-2021.lcd

Acquired by	: Admin
Sample Name	: sample 2.1_3-12-2021_Mightysil_70Hex-30EtAc-0.2aa
Sample ID	: sample 2.1_3-12-21
Tray#	: 1
Vail #	: 1
Injection Volume	: 10 uL
Data File Name	: sample 2.1_3-12-2021.lcd
Method File Name	: phenolic acid 275.lcm
Batch File Name	:
Report File Name	: ethyl ferulate wavelength 275.lcr
Data Acquired	: 3/12/2564 12:35:48
Data Processed	: 21/12/2564 10:53:28

<Chromatogram>

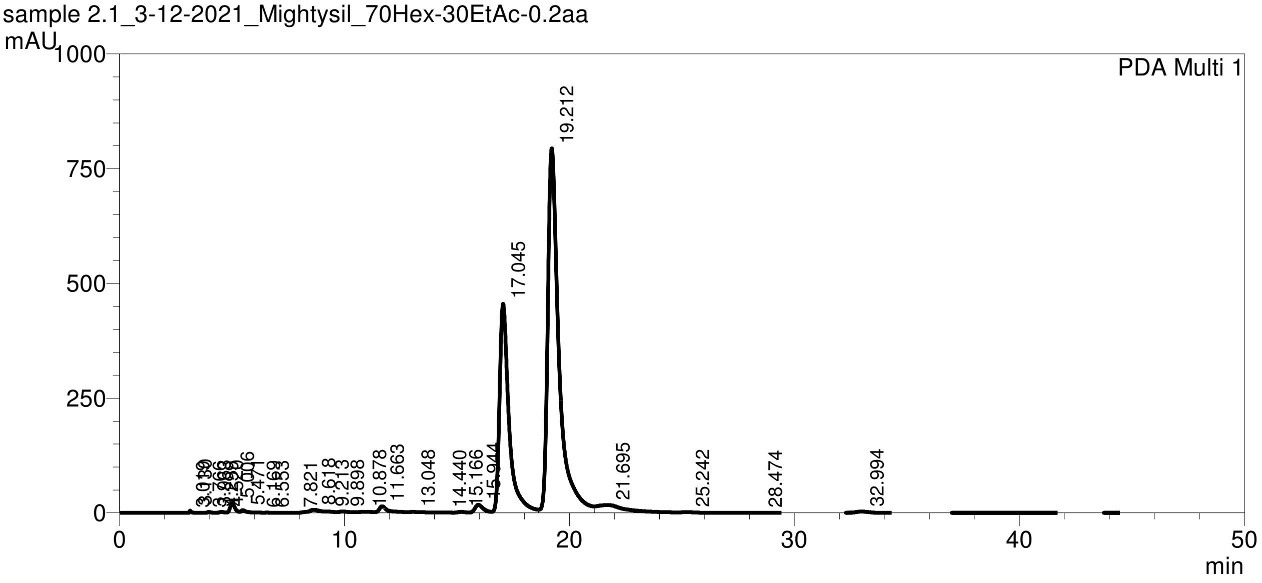

1 PDA Multi 1/300nm 4nm

PDA Ch1 300nm 4nm

Peak#	Ret. Time	Area	Height	Area %	Height %
1	3.019	5607	469	0.012	0.034
2	3.130	31451	5453	0.069	0.398
3	3.766	6506	705	0.014	0.051
4	3.963	27102	2195	0.059	0.160
5	4.299	6135	689	0.013	0.050
6	4.520	34134	2277	0.075	0.166
7	5.006	327945	21311	0.716	1.557
8	5.471	108531	6187	0.237	0.452
9	6.169	14547	875	0.032	0.064
10	6.553	12667	625	0.028	0.046
11	7.821	8042	482	0.018	0.035
12	8.618	253561	6615	0.554	0.483
13	9.213	81863	3212	0.179	0.235
14	9.898	108495	3380	0.237	0.247
15	10.878	120313	2670	0.263	0.195
16	11.663	451144	14611	0.985	1.067
17	13.048	131642	2330	0.288	0.170
18	14.440	42988	1324	0.094	0.097
19	15.166	66918	2442	0.146	0.178
20	15.944	496398	17845	1.084	1.303
21	17.045	13566203	456135	29.628	33.318
22	19.212	27774783	794734	60.659	58.050
23	21.695	1847269	17271	4.034	1.262
24	25.242	29058	802	0.063	0.059
25	28.474	22163	527	0.048	0.038
26	32.994	212701	3874	0.465	0.283
Total		45788168	1369042	100.000	100.000

==== Shimadzu LCsolution Analysis Report ====

E:\Pang\phenolic acid\2021-12-03 analyze sample\sample 2.1_3-12-2021.lcd

Acquired by	: Admin
Sample Name	: sample 2.1_3-12-2021_Mightysil_70Hex-30EtAc-0.2aa
Sample ID	: sample 2.1_3-12-21
Tray#	: 1
Vail #	: 1
Injection Volume	: 10 uL
Data File Name	: sample 2.1_3-12-2021.lcd
Method File Name	: phenolic acid 275.lcm
Batch File Name	:
Report File Name	: ethyl ferulate wavelength 275.lcr
Data Acquired	: 3/12/2564 12:35:48
Data Processed	: 21/12/2564 10:53:28

<Chromatogram>

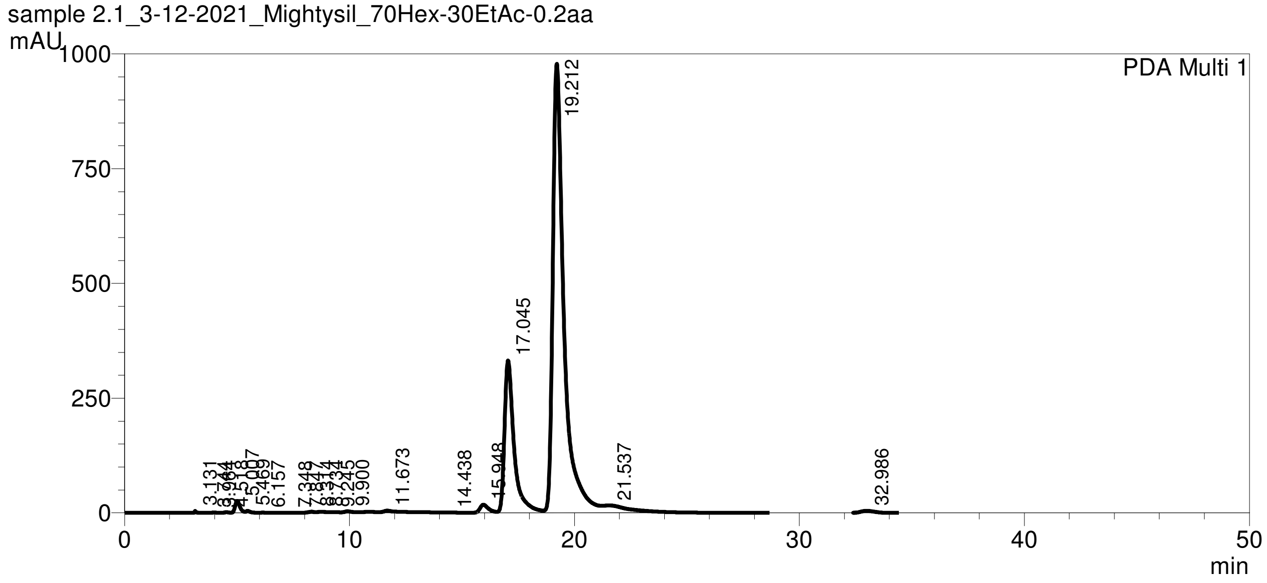

1 PDA Multi 1/320nm 4nm

PDA Ch1 320nm 4nm

Peak#	Ret. Time	Area	Height	Area %	Height %
1	3.131	24955	4941	0.054	0.357
2	3.744	2631	303	0.006	0.022
3	3.964	15812	1234	0.034	0.089
4	4.518	22023	1596	0.047	0.115
5	5.007	395858	26149	0.850	1.888
6	5.469	46542	4080	0.100	0.295
7	6.157	3353	292	0.007	0.021
8	7.348	1157	112	0.002	0.008
9	7.847	5946	379	0.013	0.027
10	8.314	39355	2314	0.084	0.167
11	8.734	48119	1832	0.103	0.132
12	9.245	15551	861	0.033	0.062
13	9.900	33820	2075	0.073	0.150
14	11.673	87007	3070	0.187	0.222
15	14.438	4716	222	0.010	0.016
16	15.948	464658	17517	0.997	1.265
17	17.045	9818616	331490	21.075	23.935
18	19.212	35087219	977907	75.313	70.610
19	21.537	131323	2821	0.282	0.204
20	32.986	339625	5737	0.729	0.414
Total		46588285	1384932	100.000	100.000

==== Shimadzu LCsolution Analysis Report ====

E:\Pang\phenolic acid\2021-12-03 analyze sample\sample 2.2_3-12-2021.lcd

Acquired by	: Admin
Sample Name	: sample 2.2_3-12-2021_Mightysil_70Hex-30EtAc-0.2aa
Sample ID	: sample 2.2_3-12-21
Tray#	: 1
Vail #	: 1
Injection Volume	: 10 uL
Data File Name	: sample 2.2_3-12-2021.lcd
Method File Name	: phenolic acid 275.lcm
Batch File Name	:
Report File Name	: ethyl ferulate wavelength 275.lcr
Data Acquired	: 3/12/2564 14:15:22
Data Processed	: 21/12/2564 10:55:04

<Chromatogram>

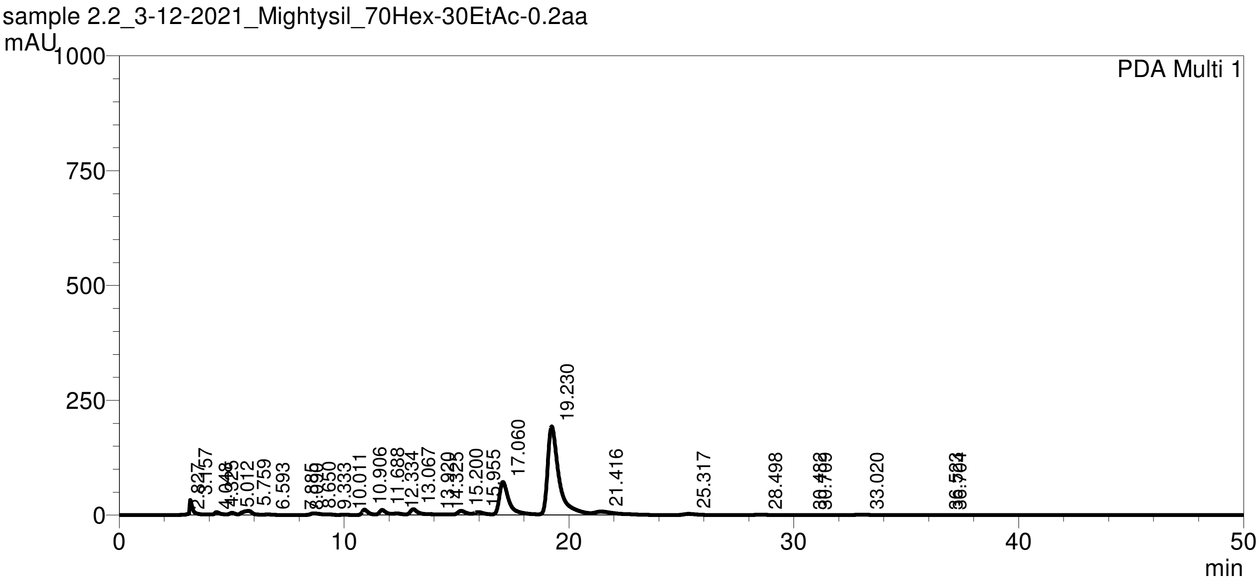

1 PDA Multi 1/257nm 4nm

PDA Ch1 257nm 4nm

Peak#	Ret. Time	Area	Height	Area %	Height %
1	2.827	5193	439	0.044	0.114
2	3.157	367583	32843	3.134	8.510
3	4.048	3029	338	0.026	0.088
4	4.325	109467	6433	0.933	1.667
5	5.012	97646	4753	0.833	1.232
6	5.759	291773	8939	2.488	2.316
7	6.593	38755	1734	0.330	0.449
8	7.885	3407	253	0.029	0.065
9	8.090	2208	346	0.019	0.090
10	8.650	115653	3711	0.986	0.962
11	9.333	26575	1363	0.227	0.353
12	10.011	15085	691	0.129	0.179
13	10.906	211030	11431	1.799	2.962
14	11.688	220457	10543	1.880	2.732
15	12.334	72581	2949	0.619	0.764
16	13.067	283375	11819	2.416	3.062
17	13.920	12779	734	0.109	0.190
18	14.325	1239	250	0.011	0.065
19	15.200	193607	8084	1.651	2.095
20	15.955	156331	5577	1.333	1.445
21	17.060	2117826	71274	18.056	18.468
22	19.230	7023765	192425	59.883	49.859
23	21.416	165756	4124	1.413	1.068
24	25.317	114633	2690	0.977	0.697
25	28.498	35579	769	0.303	0.199
26	30.482	7148	286	0.061	0.074
27	30.709	2007	214	0.017	0.055
28	33.020	31911	734	0.272	0.190
29	36.523	1259	95	0.011	0.025

Peak#	Ret. Time	Area	Height	Area %	Height %
30	36.704	1403	96	0.012	0.025
Total		11729059	385937	100.000	100.000

==== Shimadzu LCsolution Analysis Report ====

E:\Pang\phenolic acid\2021-12-03 analyze sample\sample 2.2_3-12-2021.lcd

Acquired by	: Admin
Sample Name	: sample 2.2_3-12-2021_Mightysil_70Hex-30EtAc-0.2aa
Sample ID	: sample 2.2_3-12-21
Tray#	: 1
Vail #	: 1
Injection Volume	: 10 uL
Data File Name	: sample 2.2_3-12-2021.lcd
Method File Name	: phenolic acid 275.lcm
Batch File Name	:
Report File Name	: ethyl ferulate wavelength 275.lcr
Data Acquired	: 3/12/2564 14:15:22
Data Processed	: 21/12/2564 10:55:04

<Chromatogram>

sample 2.2_3-12-2021_Mightysil_70Hex-30EtAc-0.2aa

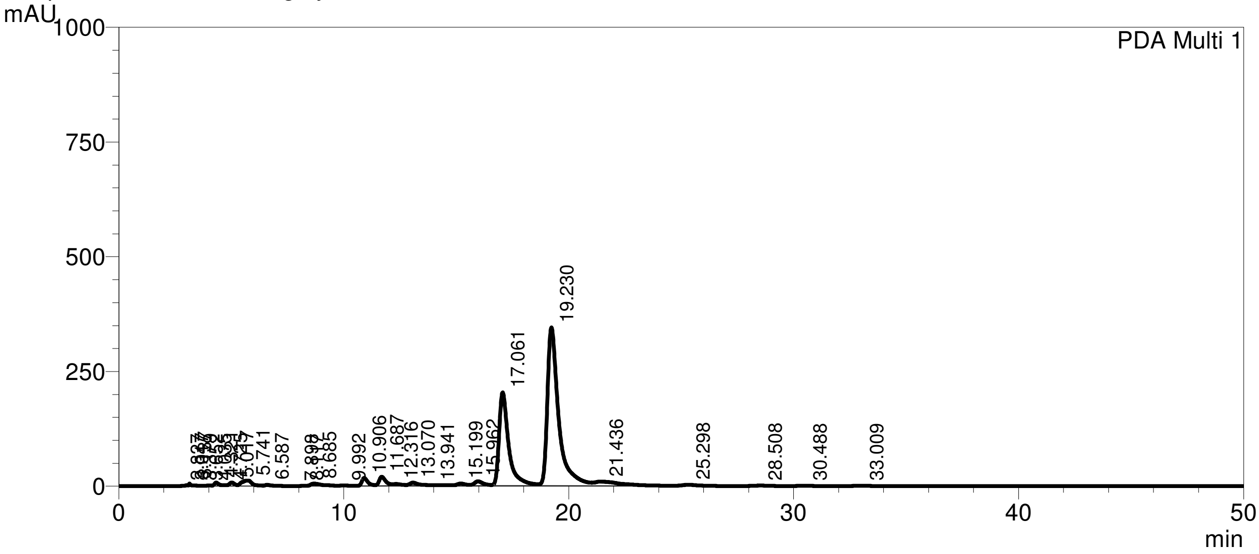

1 PDA Multi 1/271nm 4nm

PDA Ch1 271nm 4nm

Peak#	Ret. Time	Area	Height	Area %	Height %
1	2.827	3082	328	0.014	0.049
2	3.017	15129	1999	0.071	0.301
3	3.137	30588	5181	0.144	0.780
4	3.379	13231	1034	0.062	0.156
5	3.652	11032	759	0.052	0.114
6	4.035	19612	1423	0.092	0.214
7	4.321	114709	7785	0.540	1.172
8	4.725	13004	1710	0.061	0.257
9	5.017	132689	7850	0.624	1.182
10	5.741	397277	12024	1.869	1.811
11	6.587	61509	2591	0.289	0.390
12	7.898	1265	167	0.006	0.025
13	8.117	2446	331	0.012	0.050
14	8.685	195079	5237	0.918	0.789
15	9.992	23823	1132	0.112	0.170
16	10.906	311688	17170	1.466	2.586
17	11.687	403685	19401	1.899	2.922
18	12.316	82904	3366	0.390	0.507
19	13.070	153679	5975	0.723	0.900
20	13.941	5037	377	0.024	0.057
21	15.199	88826	3856	0.418	0.581
22	15.962	230125	8718	1.082	1.313
23	17.061	5990192	202813	28.176	30.545
24	19.230	12537482	344249	58.973	51.846
25	21.436	199846	3722	0.940	0.561
26	25.298	103495	2257	0.487	0.340
27	28.508	55945	1048	0.263	0.158
28	30.488	34147	798	0.161	0.120
29	33.009	28073	680	0.132	0.102

Peak#	Ret. Time	Area	Height	Area %	Height %
Total		21259602	663980	100.000	100.000

==== Shimadzu LCsolution Analysis Report ====

E:\Pang\phenolic acid\2021-12-03 analyze sample\sample 2.2_3-12-2021.lcd

Acquired by : Admin
Sample Name : sample 2.2_3-12-2021_Mightysil_70Hex-30EtAc-0.2aa
Sample ID : sample 2.2_3-12-21
Tray# : 1
Vail # : 1
Injection Volume : 10 uL
Data File Name : sample 2.2_3-12-2021.lcd
Method File Name : phenolic acid 275.lcm
Batch File Name :
Report File Name : ethyl ferulate wavelength 275.lcr
Data Acquired : 3/12/2564 14:15:22
Data Processed : 21/12/2564 10:55:04

<Chromatogram>

sample 2.2_3-12-2021_Mightysil_70Hex-30EtAc-0.2aa

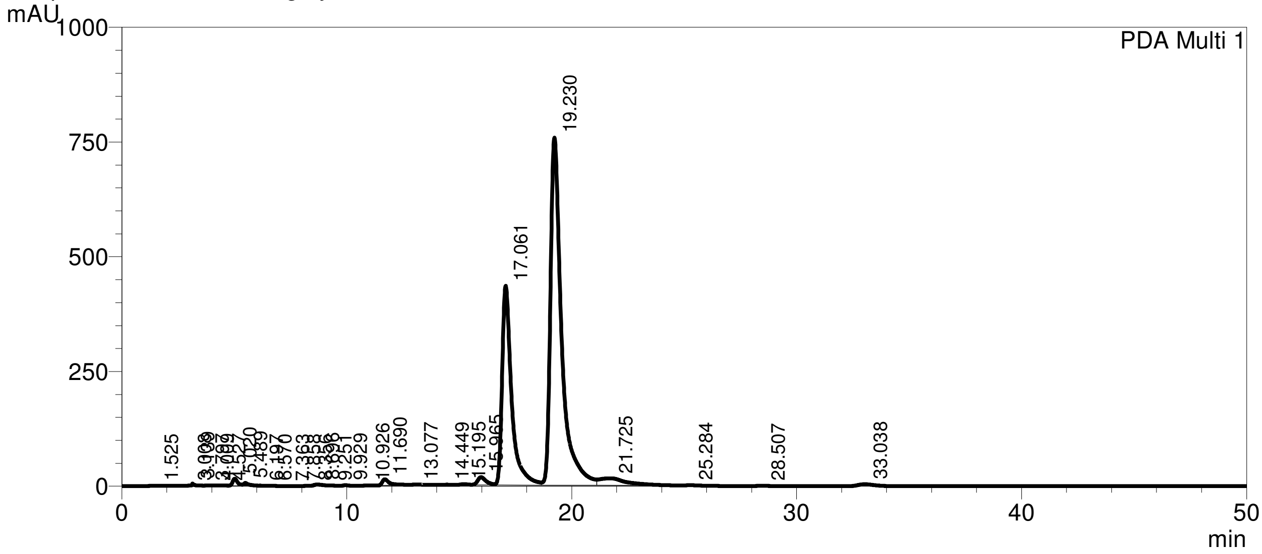

1 PDA Multi 1/300nm 4nm

PDA Ch1 300nm 4nm

Peak#	Ret. Time	Area	Height	Area %	Height %
1	1.525	2968	27	0.007	0.002
2	3.008	1303	173	0.003	0.014
3	3.139	31851	4713	0.076	0.368
4	3.797	3163	293	0.008	0.023
5	4.009	10970	685	0.026	0.053
6	4.527	14085	805	0.034	0.063
7	5.020	240994	15420	0.573	1.203
8	5.489	104947	6079	0.250	0.474
9	6.197	1489	171	0.004	0.013
10	6.570	5125	342	0.012	0.027
11	7.363	1656	137	0.004	0.011
12	7.858	1187	131	0.003	0.010
13	8.356	13451	897	0.032	0.070
14	8.696	94724	3195	0.225	0.249
15	9.251	26853	1440	0.064	0.112
16	9.929	26182	1620	0.062	0.126
17	10.926	14186	688	0.034	0.054
18	11.690	313843	13327	0.747	1.040
19	13.077	17939	801	0.043	0.062
20	14.449	7552	383	0.018	0.030
21	15.195	37752	1735	0.090	0.135
22	15.965	442326	16831	1.053	1.313
23	17.061	12788161	434179	30.431	33.873
24	19.230	26255221	757437	62.477	59.092
25	21.725	1287620	14788	3.064	1.154
26	25.284	22758	679	0.054	0.053
27	28.507	24067	491	0.057	0.038
28	33.038	231469	4332	0.551	0.338
Total		42023843	1281800	100.000	100.000

==== Shimadzu LCsolution Analysis Report ====

E:\Pang\phenolic acid\2021-12-03 analyze sample\sample 2.2_3-12-2021.lcd

Acquired by : Admin
Sample Name : sample 2.2_3-12-2021_Mightysil_70Hex-30EtAc-0.2aa
Sample ID : sample 2.2_3-12-21
Tray# : 1
Vail # : 1
Injection Volume : 10 uL
Data File Name : sample 2.2_3-12-2021.lcd
Method File Name : phenolic acid 275.lcm
Batch File Name :
Report File Name : ethyl ferulate wavelength 275.lcr
Data Acquired : 3/12/2564 14:15:22
Data Processed : 21/12/2564 10:55:04

<Chromatogram>

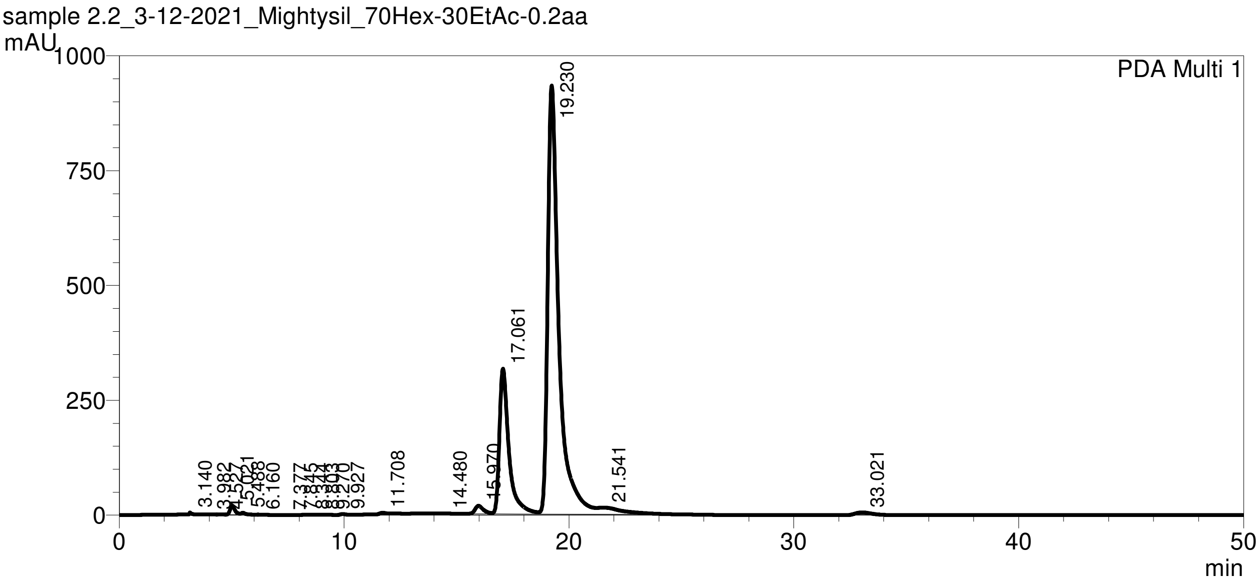

1 PDA Multi 1/320nm 4nm

PDA Ch1 320nm 4nm

Peak#	Ret. Time	Area	Height	Area %	Height %
1	3.140	27953	4573	0.063	0.349
2	3.982	3258	246	0.007	0.019
3	4.527	5629	512	0.013	0.039
4	5.021	289961	19062	0.658	1.454
5	5.488	46982	3962	0.107	0.302
6	6.160	2762	240	0.006	0.018
7	7.377	2568	200	0.006	0.015
8	7.845	2153	190	0.005	0.015
9	8.344	14885	929	0.034	0.071
10	8.803	30541	950	0.069	0.072
11	9.270	12046	715	0.027	0.055
12	9.927	31197	2014	0.071	0.154
13	11.708	41316	2476	0.094	0.189
14	14.480	6512	252	0.015	0.019
15	15.970	450722	17192	1.023	1.311
16	17.061	9313882	316102	21.132	24.105
17	19.230	33288629	932693	75.526	71.123
18	21.541	124176	2621	0.282	0.200
19	33.021	380607	6449	0.864	0.492
Total		44075778	1311377	100.000	100.000

==== Shimadzu LCsolution Analysis Report ====

E:\Pang\phenolic acid\2021-12-03 analyze sample\sample 3.1_6-12-2021.lcd

Acquired by	: Admin
Sample Name	: sample 3.1_6-12-2021_Mightysil_70Hex-30EtAc-0.2aa
Sample ID	: sample 3.1_6-12-21
Tray#	: 1
Vail #	: 1
Injection Volume	: 10 uL
Data File Name	: sample 3.1_6-12-2021.lcd
Method File Name	: phenolic acid 275.lcm
Batch File Name	:
Report File Name	: ethyl ferulate wavelength 275.lcr
Data Acquired	: 6/12/2564 9:25:09
Data Processed	: 21/12/2564 10:56:25

<Chromatogram>

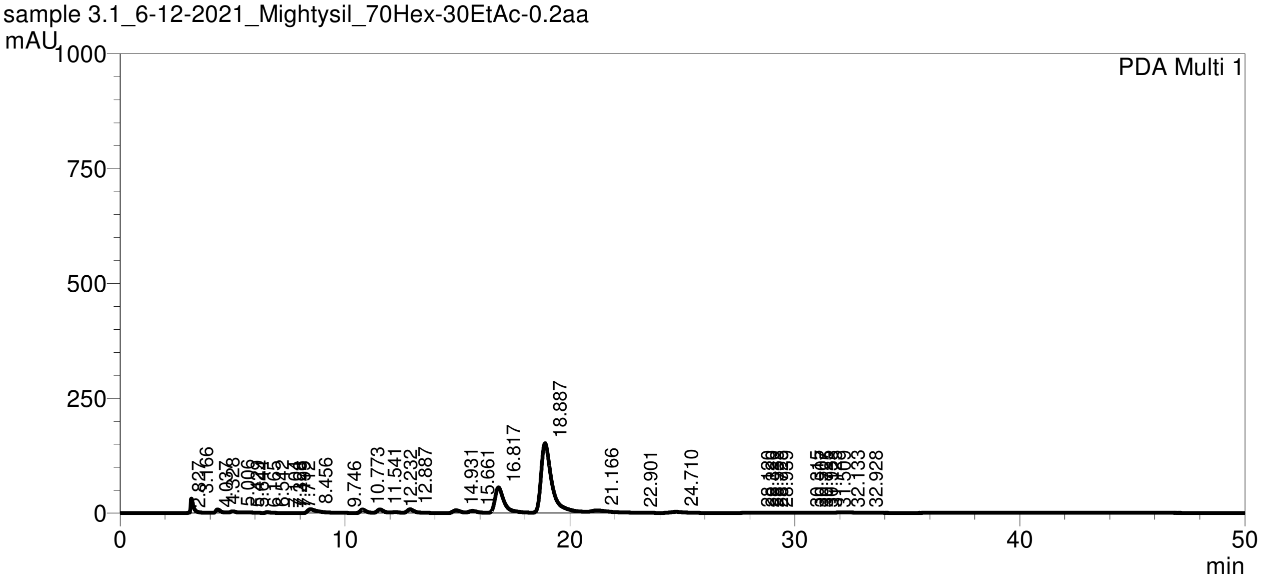

1 PDA Multi 1/257nm 4nm

PDA Ch1 257nm 4nm

Peak#	Ret. Time	Area	Height	Area %	Height %
1	2.827	2540	263	0.028	0.083
2	3.166	332373	31957	3.634	10.114
3	4.037	2119	258	0.023	0.082
4	4.328	141250	8254	1.545	2.612
5	5.006	80778	4041	0.883	1.279
6	5.429	14517	1740	0.159	0.551
7	5.644	50587	1792	0.553	0.567
8	6.165	12140	694	0.133	0.220
9	6.542	42795	2221	0.468	0.703
10	7.104	7759	531	0.085	0.168
11	7.328	2147	331	0.023	0.105
12	7.499	5460	349	0.060	0.110
13	7.712	4307	421	0.047	0.133
14	8.456	320586	8446	3.506	2.673
15	9.746	4719	160	0.052	0.051
16	10.773	149580	7781	1.636	2.463
17	11.541	165761	8157	1.813	2.582
18	12.232	56450	2229	0.617	0.706
19	12.887	204752	8169	2.239	2.586
20	14.931	139543	5983	1.526	1.894
21	15.661	129102	4578	1.412	1.449
22	16.817	1607513	55814	17.578	17.665
23	18.887	5339258	151849	58.383	48.060
24	21.166	143188	3416	1.566	1.081
25	22.901	1174	53	0.013	0.017

Peak#	Ret. Time	Area	Height	Area %	Height %
26	24.710	88779	2251	0.971	0.712
27	28.120	17753	487	0.194	0.154
28	28.320	2466	377	0.027	0.119
29	28.533	3743	310	0.041	0.098
30	28.725	3661	275	0.040	0.087
31	28.939	1426	172	0.016	0.054
32	30.315	1969	161	0.022	0.051
33	30.507	2263	224	0.025	0.071
34	30.725	2088	277	0.023	0.088
35	30.933	2873	255	0.031	0.081
36	31.125	2632	209	0.029	0.066
37	31.509	3723	220	0.041	0.070
38	32.133	49889	1026	0.546	0.325
39	32.928	1531	223	0.017	0.071
Total		9145195	315957	100.000	100.000

==== Shimadzu LCsolution Analysis Report ====

E:\Pang\phenolic acid\2021-12-03 analyze sample\sample 3.1_6-12-2021.lcd

Acquired by	: Admin
Sample Name	: sample 3.1_6-12-2021_Mightysil_70Hex-30EtAc-0.2aa
Sample ID	: sample 3.1_6-12-21
Tray#	: 1
Vail #	: 1
Injection Volume	: 10 uL
Data File Name	: sample 3.1_6-12-2021.lcd
Method File Name	: phenolic acid 275.lcm
Batch File Name	:
Report File Name	: ethyl ferulate wavelength 275.lcr
Data Acquired	: 6/12/2564 9:25:09
Data Processed	: 21/12/2564 10:56:25

<Chromatogram>

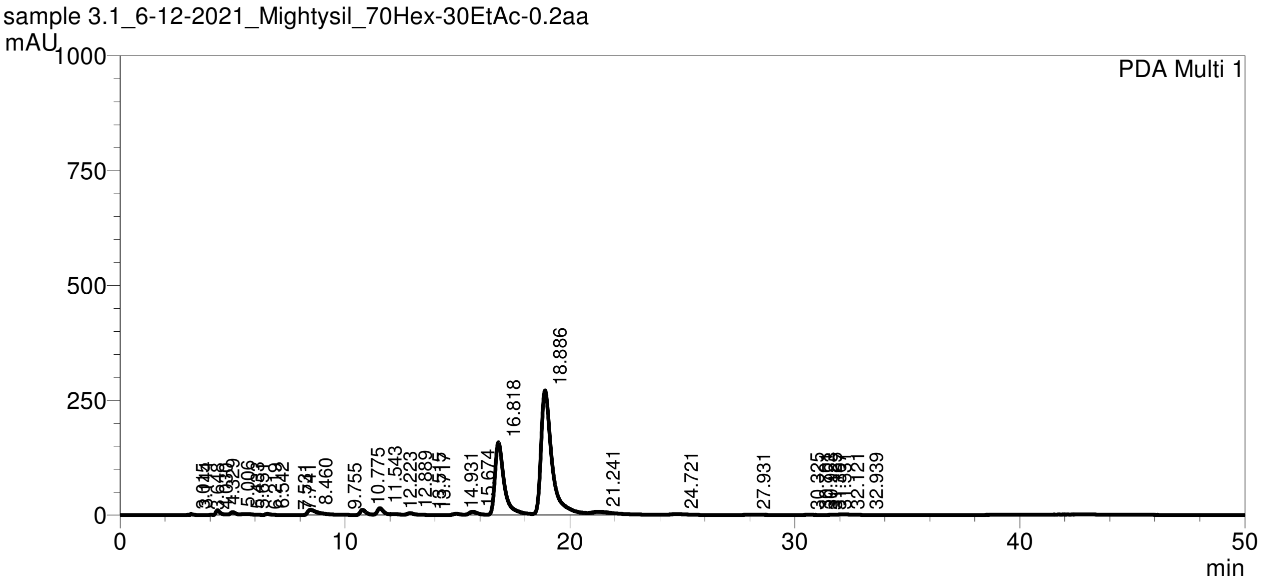

1 PDA Multi 1/271nm 4nm

PDA Ch1 271nm 4nm

Peak#	Ret. Time	Area	Height	Area %	Height %
1	3.015	7854	770	0.048	0.147
2	3.144	24055	2780	0.148	0.531
3	3.648	5074	409	0.031	0.078
4	4.030	11334	856	0.070	0.163
5	4.329	158884	10519	0.978	2.010
6	5.006	116255	6505	0.715	1.243
7	5.433	18620	2136	0.115	0.408
8	5.651	57952	2201	0.357	0.421
9	6.219	10755	605	0.066	0.116
10	6.542	51195	2742	0.315	0.524
11	7.531	1270	121	0.008	0.023
12	7.741	2908	236	0.018	0.045
13	8.460	402468	11562	2.477	2.209
14	9.755	29572	1014	0.182	0.194
15	10.775	211704	11535	1.303	2.204
16	11.543	300382	14837	1.849	2.835
17	12.223	52738	2179	0.325	0.416
18	12.889	90648	3962	0.558	0.757
19	13.515	2779	351	0.017	0.067
20	13.717	2760	227	0.017	0.043
21	14.931	60741	2815	0.374	0.538
22	15.674	195005	7161	1.200	1.368
23	16.818	4543483	158426	27.963	30.273
24	18.886	9554230	271205	58.801	51.824
25	21.241	168549	3158	1.037	0.603

Peak#	Ret. Time	Area	Height	Area %	Height %
26	24.721	78338	1870	0.482	0.357
27	27.931	12029	386	0.074	0.074
28	30.325	1601	214	0.010	0.041
29	30.701	6767	316	0.042	0.060
30	30.923	3412	252	0.021	0.048
31	31.125	1531	175	0.009	0.033
32	31.307	2188	149	0.013	0.028
33	31.531	1499	261	0.009	0.050
34	32.121	57748	1155	0.355	0.221
35	32.939	2048	232	0.013	0.044
Total		16248375	523322	100.000	100.000

==== Shimadzu LCsolution Analysis Report ====

E:\Pang\phenolic acid\2021-12-03 analyze sample\sample 3.1_6-12-2021.lcd

Acquired by	: Admin
Sample Name	: sample 3.1_6-12-2021_Mightysil_70Hex-30EtAc-0.2aa
Sample ID	: sample 3.1_6-12-21
Tray#	: 1
Vail #	: 1
Injection Volume	: 10 uL
Data File Name	: sample 3.1_6-12-2021.lcd
Method File Name	: phenolic acid 275.lcm
Batch File Name	:
Report File Name	: ethyl ferulate wavelength 275.lcr
Data Acquired	: 6/12/2564 9:25:09
Data Processed	: 21/12/2564 10:56:25

<Chromatogram>

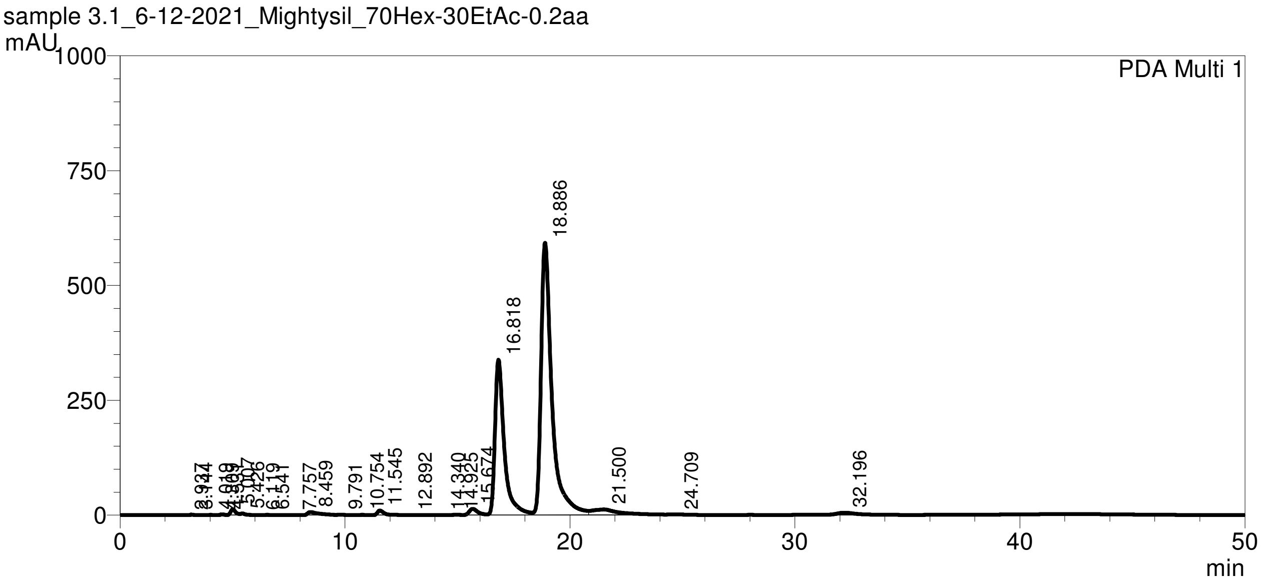

1 PDA Multi 1/300nm 4nm

PDA Ch1 300nm 4nm

Peak#	Ret. Time	Area	Height	Area %	Height %
1	2.937	1648	274	0.005	0.027
2	3.144	13600	1928	0.042	0.192
3	4.019	6267	530	0.019	0.053
4	4.309	1298	202	0.004	0.020
5	4.531	20885	1683	0.065	0.167
6	5.007	202047	12781	0.627	1.272
7	5.426	57380	3914	0.178	0.389
8	6.119	8172	460	0.025	0.046
9	6.541	9573	624	0.030	0.062
10	7.757	1332	131	0.004	0.013
11	8.459	261460	6909	0.812	0.687
12	9.791	32473	1346	0.101	0.134
13	10.754	21365	759	0.066	0.075
14	11.545	234027	10242	0.726	1.019
15	12.892	7260	414	0.023	0.041
16	14.340	4545	278	0.014	0.028
17	14.925	24621	1216	0.076	0.121
18	15.674	369475	13735	1.147	1.367
19	16.818	9688620	338324	30.076	33.662
20	18.886	20023439	592584	62.157	58.961
21	21.500	950538	11569	2.951	1.151
22	24.709	23032	670	0.071	0.067
23	32.196	251161	4480	0.780	0.446
Total		32214221	1005051	100.000	100.000

==== Shimadzu LCsolution Analysis Report ====

E:\Pang\phenolic acid\2021-12-03 analyze sample\sample 3.1_6-12-2021.lcd

Acquired by	: Admin
Sample Name	: sample 3.1_6-12-2021_Mightysil_70Hex-30EtAc-0.2aa
Sample ID	: sample 3.1_6-12-21
Tray#	: 1
Vail #	: 1
Injection Volume	: 10 uL
Data File Name	: sample 3.1_6-12-2021.lcd
Method File Name	: phenolic acid 275.lcm
Batch File Name	:
Report File Name	: ethyl ferulate wavelength 275.lcr
Data Acquired	: 6/12/2564 9:25:09
Data Processed	: 21/12/2564 10:56:25

<Chromatogram>

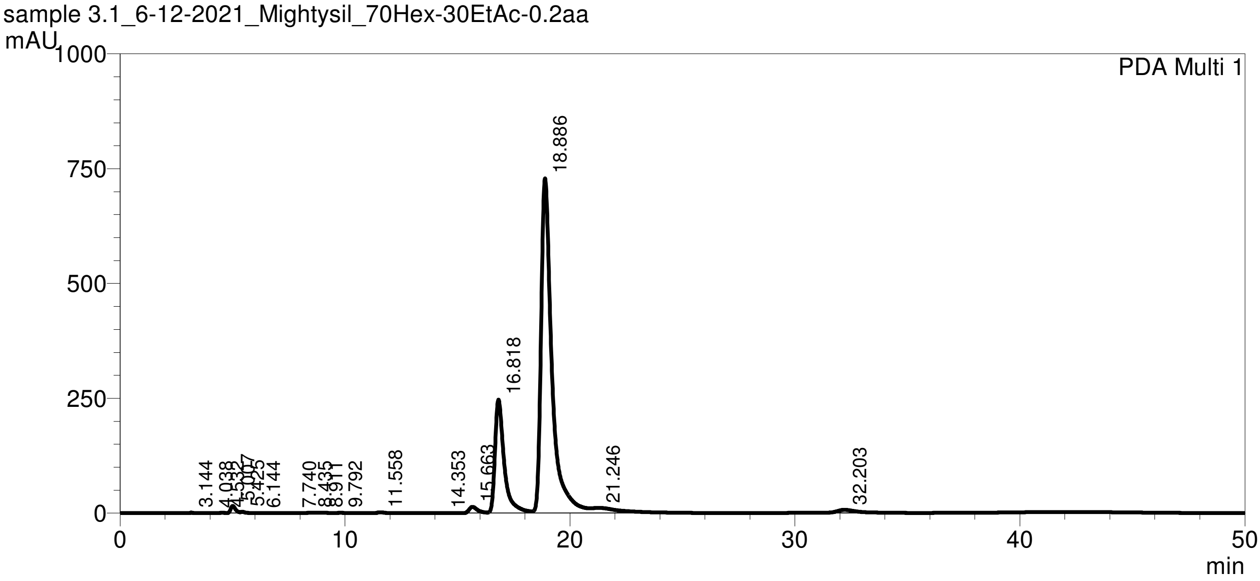

1 PDA Multi 1/320nm 4nm

PDA Ch1 320nm 4nm

Peak#	Ret. Time	Area	Height	Area %	Height %
1	3.144	10698	1717	0.032	0.167
2	4.038	2801	175	0.008	0.017
3	4.532	15484	1314	0.046	0.128
4	5.007	247000	15938	0.732	1.552
5	5.425	42165	3105	0.125	0.302
6	6.144	8185	415	0.024	0.040
7	7.740	2141	185	0.006	0.018
8	8.435	27166	1308	0.080	0.127
9	8.911	46081	1282	0.137	0.125
10	9.792	21640	1229	0.064	0.120
11	11.558	39100	2063	0.116	0.201
12	14.353	3291	192	0.010	0.019
13	15.663	381108	13999	1.129	1.363
14	16.818	7096048	247189	21.020	24.064
15	18.886	25319478	728612	75.002	70.930
16	21.246	117658	2156	0.349	0.210
17	32.203	378154	6343	1.120	0.617
Total		33758196	1027222	100.000	100.000

==== Shimadzu LCsolution Analysis Report ====

E:\Pang\phenolic acid\2021-12-03 analyze sample\sample 3.2_6-12-2021.lcd

Acquired by	: Admin
Sample Name	: sample 3.2_6-12-2021_Mightysil_70Hex-30EtAc-0.2aa
Sample ID	: sample 3.2_6-12-21
Tray#	: 1
Vail #	: 1
Injection Volume	: 10 uL
Data File Name	: sample 3.2_6-12-2021.lcd
Method File Name	: phenolic acid 275.lcm
Batch File Name	:
Report File Name	: ethyl ferulate wavelength 275.lcr
Data Acquired	: 6/12/2564 11:03:20
Data Processed	: 9/12/2564 12:02:17

<Chromatogram>

sample 3.2_6-12-2021_Mightysil_70Hex-30EtAc-0.2aa

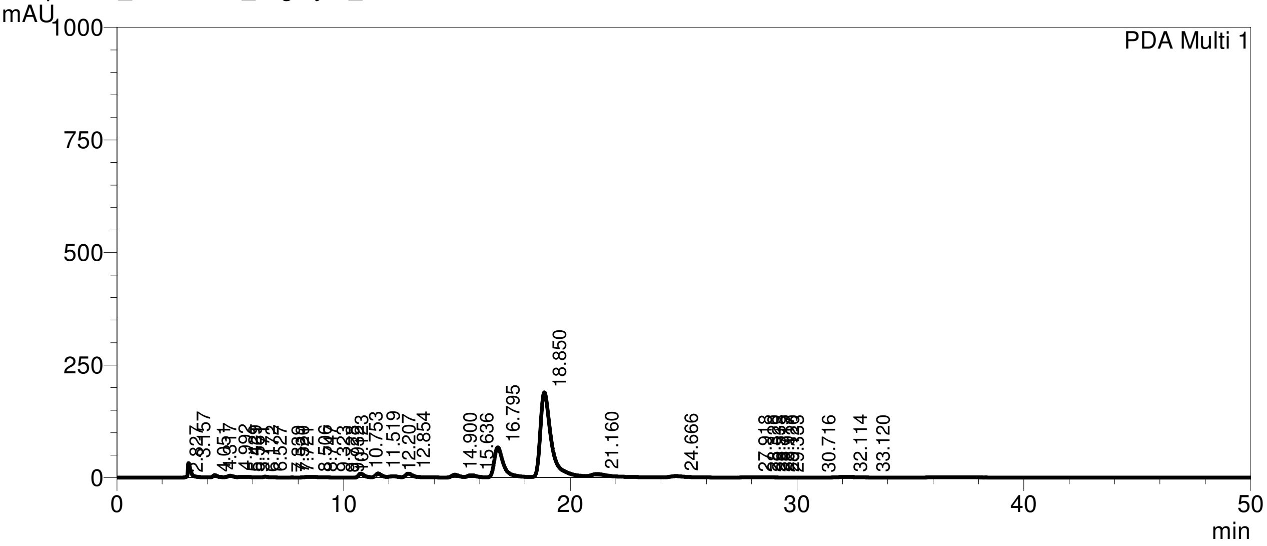

1 PDA Multi 1/257nm 4nm

PDA Ch1 257nm 4nm

Peak#	Ret. Time	Area	Height	Area %	Height %
1	2.827	4693	393	0.044	0.107
2	3.157	352529	32511	3.306	8.878
3	4.051	1780	213	0.017	0.058
4	4.317	88995	5548	0.835	1.515
5	4.992	86301	4154	0.809	1.134
6	5.429	17257	1759	0.162	0.480
7	5.561	22523	1916	0.211	0.523
8	5.771	29208	1774	0.274	0.485
9	6.172	16663	1002	0.156	0.274
10	6.527	48019	2149	0.450	0.587
11	7.339	1989	256	0.019	0.070
12	7.520	3701	358	0.035	0.098
13	7.721	6256	458	0.059	0.125
14	8.506	40751	1488	0.382	0.406
15	8.747	24664	1246	0.231	0.340
16	9.323	8698	501	0.082	0.137
17	9.728	4321	288	0.041	0.079
18	9.932	3442	327	0.032	0.089
19	10.123	1554	222	0.015	0.061
20	10.753	159554	8640	1.496	2.359

Peak#	Ret. Time	Area	Height	Area %	Height %
21	11.519	172644	8903	1.619	2.431
22	12.207	79291	2878	0.744	0.786
23	12.854	214473	8704	2.011	2.377
24	14.900	137743	6091	1.292	1.663
25	15.636	144350	5091	1.354	1.390
26	16.795	1919174	66817	17.997	18.247
27	18.850	6624966	188679	62.124	51.527
28	21.160	195004	4939	1.829	1.349
29	24.666	112215	2775	1.052	0.758
30	27.918	29072	809	0.273	0.221
31	28.320	6201	581	0.058	0.159
32	28.533	2955	481	0.028	0.131
33	28.715	6800	424	0.064	0.116
34	28.917	2746	306	0.026	0.084
35	29.120	1145	196	0.011	0.054
36	29.333	1253	124	0.012	0.034
37	30.716	1072	79	0.010	0.022
38	32.114	70182	1431	0.658	0.391
39	33.120	1893	177	0.018	0.048
40	54.507	1085	76	0.010	0.021
41	54.720	1757	154	0.016	0.042
42	54.923	2034	210	0.019	0.057
43	55.104	3052	244	0.029	0.067
44	55.517	4304	259	0.040	0.071
45	55.723	2395	238	0.022	0.065
46	55.936	1986	172	0.019	0.047
47	56.128	1373	134	0.013	0.037
Total		10664066	366175	100.000	100.000

==== Shimadzu LCsolution Analysis Report ====

E:\Pang\phenolic acid\2021-12-03 analyze sample\sample 3.2_6-12-2021.lcd

Acquired by	: Admin
Sample Name	: sample 3.2_6-12-2021_Mightysil_70Hex-30EtAc-0.2aa
Sample ID	: sample 3.2_6-12-21
Tray#	: 1
Vail #	: 1
Injection Volume	: 10 uL
Data File Name	: sample 3.2_6-12-2021.lcd
Method File Name	: phenolic acid 275.lcm
Batch File Name	:
Report File Name	: ethyl ferulate wavelength 275.lcr
Data Acquired	: 6/12/2564 11:03:20
Data Processed	: 9/12/2564 12:02:17

<Chromatogram>

sample 3.2_6-12-2021_Mightysil_70Hex-30EtAc-0.2aa

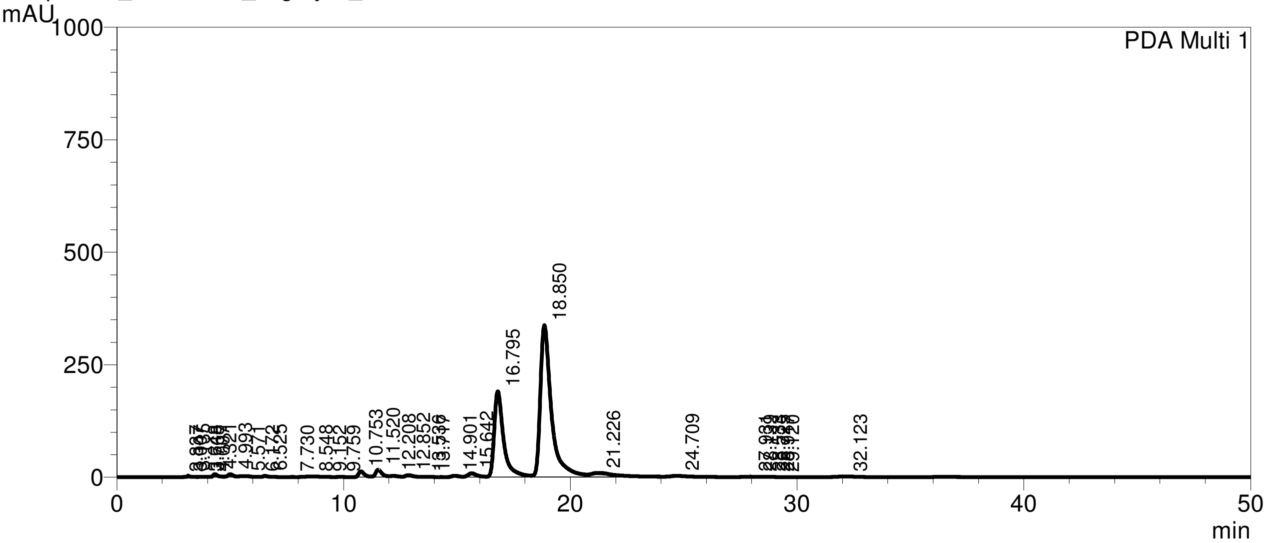

1 PDA Multi 1/271nm 4nm

PDA Ch1 271nm 4nm

Peak#	Ret. Time	Area	Height	Area %	Height %
1	2.837	4013	372	0.021	0.060
2	3.007	9358	1163	0.049	0.189
3	3.135	32693	3705	0.170	0.601
4	3.648	5333	718	0.028	0.117
5	3.768	8257	794	0.043	0.129
6	4.007	12067	902	0.063	0.146
7	4.321	101485	6825	0.527	1.108
8	4.993	116612	6496	0.605	1.054
9	5.571	77811	2264	0.404	0.367
10	6.172	13829	942	0.072	0.153
11	6.525	45967	2599	0.239	0.422
12	7.730	2655	247	0.014	0.040
13	8.548	63996	1626	0.332	0.264
14	9.152	11356	580	0.059	0.094
15	9.759	9681	378	0.050	0.061
16	10.753	233609	12980	1.213	2.107
17	11.520	327313	16392	1.699	2.661
18	12.208	65230	2574	0.339	0.418
19	12.852	100660	4307	0.523	0.699
20	13.536	3700	364	0.019	0.059

Peak#	Ret. Time	Area	Height	Area %	Height %
21	13.717	2076	253	0.011	0.041
22	14.901	61270	2863	0.318	0.465
23	15.642	220346	8171	1.144	1.326
24	16.795	5450782	190288	28.302	30.888
25	18.850	11849251	336964	61.524	54.697
26	21.226	222948	4667	1.158	0.758
27	24.709	80348	2016	0.417	0.327
28	27.931	18995	887	0.099	0.144
29	28.139	16798	770	0.087	0.125
30	28.533	8597	506	0.045	0.082
31	28.725	4331	442	0.022	0.072
32	28.917	2775	308	0.014	0.050
33	29.120	1063	163	0.006	0.026
34	32.123	74418	1525	0.386	0.247
Total		19259623	616054	100.000	100.000

==== Shimadzu LCsolution Analysis Report ====

E:\Pang\phenolic acid\2021-12-03 analyze sample\sample 3.2_6-12-2021.lcd

Acquired by	: Admin
Sample Name	: sample 3.2_6-12-2021_Mightysil_70Hex-30EtAc-0.2aa
Sample ID	: sample 3.2_6-12-21
Tray#	: 1
Vail #	: 1
Injection Volume	: 10 uL
Data File Name	: sample 3.2_6-12-2021.lcd
Method File Name	: phenolic acid 275.lcm
Batch File Name	:
Report File Name	: ethyl ferulate wavelength 275.lcr
Data Acquired	: 6/12/2564 11:03:20
Data Processed	: 9/12/2564 12:02:17

<Chromatogram>

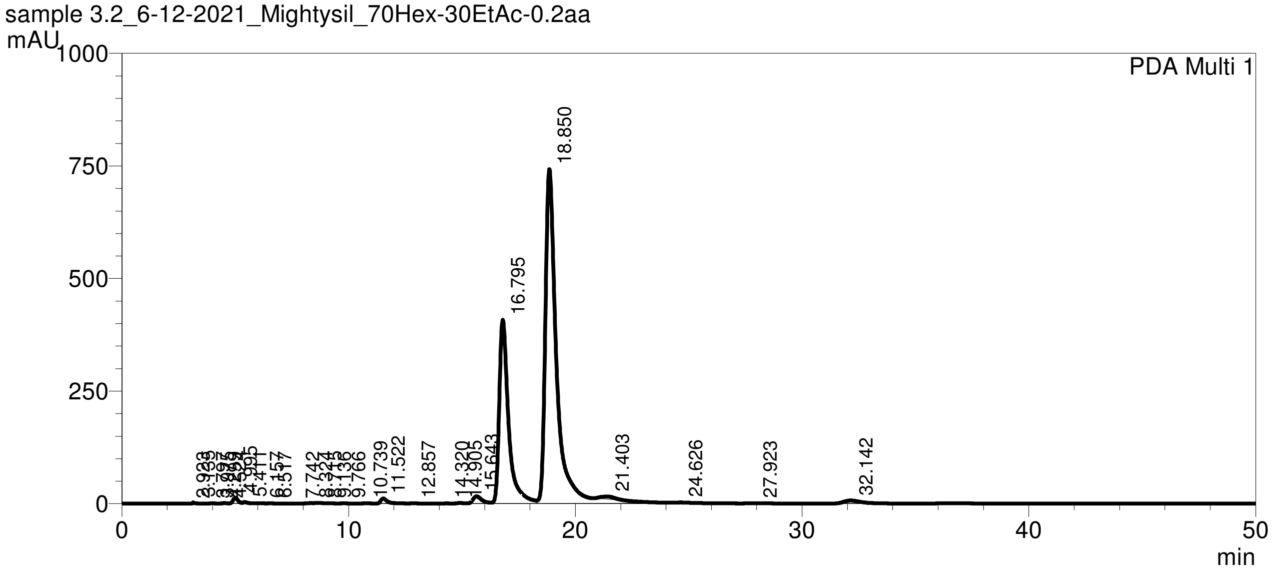

1 PDA Multi 1/300nm 4nm

PDA Ch1 300nm 4nm

Peak#	Ret. Time	Area	Height	Area %	Height %
1	2.923	1044	150	0.003	0.012
2	3.135	20802	2799	0.052	0.229
3	3.797	4853	420	0.012	0.034
4	3.975	11032	778	0.028	0.064
5	4.299	3171	336	0.008	0.028
6	4.524	16406	1217	0.041	0.100
7	4.995	202518	13191	0.509	1.081
8	5.411	52973	3499	0.133	0.287
9	6.157	14295	653	0.036	0.054
10	6.517	10938	666	0.027	0.055
11	7.742	3266	270	0.008	0.022
12	8.324	5084	341	0.013	0.028
13	8.715	3347	251	0.008	0.021
14	9.136	2560	155	0.006	0.013
15	9.766	12106	727	0.030	0.060
16	10.739	15900	671	0.040	0.055
17	11.522	242495	11226	0.609	0.920
18	12.857	8832	467	0.022	0.038
19	14.320	6428	383	0.016	0.031
20	14.905	25581	1283	0.064	0.105

Peak#	Ret. Time	Area	Height	Area %	Height %
21	15.643	440379	15970	1.106	1.309
22	16.795	11705919	407928	29.412	33.433
23	18.850	26271075	742362	66.007	60.843
24	21.403	288880	6299	0.726	0.516
25	24.626	31775	847	0.080	0.069
26	27.923	15123	432	0.038	0.035
27	32.142	383431	6816	0.963	0.559
Total		39800211	1220137	100.000	100.000

==== Shimadzu LCsolution Analysis Report ====

E:\Pang\phenolic acid\2021-12-03 analyze sample\sample 3.2_6-12-2021.lcd

Acquired by	: Admin
Sample Name	: sample 3.2_6-12-2021_Mightysil_70Hex-30EtAc-0.2aa
Sample ID	: sample 3.2_6-12-21
Tray#	: 1
Vail #	: 1
Injection Volume	: 10 uL
Data File Name	: sample 3.2_6-12-2021.lcd
Method File Name	: phenolic acid 275.lcm
Batch File Name	:
Report File Name	: ethyl ferulate wavelength 275.lcr
Data Acquired	: 6/12/2564 11:03:20
Data Processed	: 21/12/2564 11:59:56

<Chromatogram>

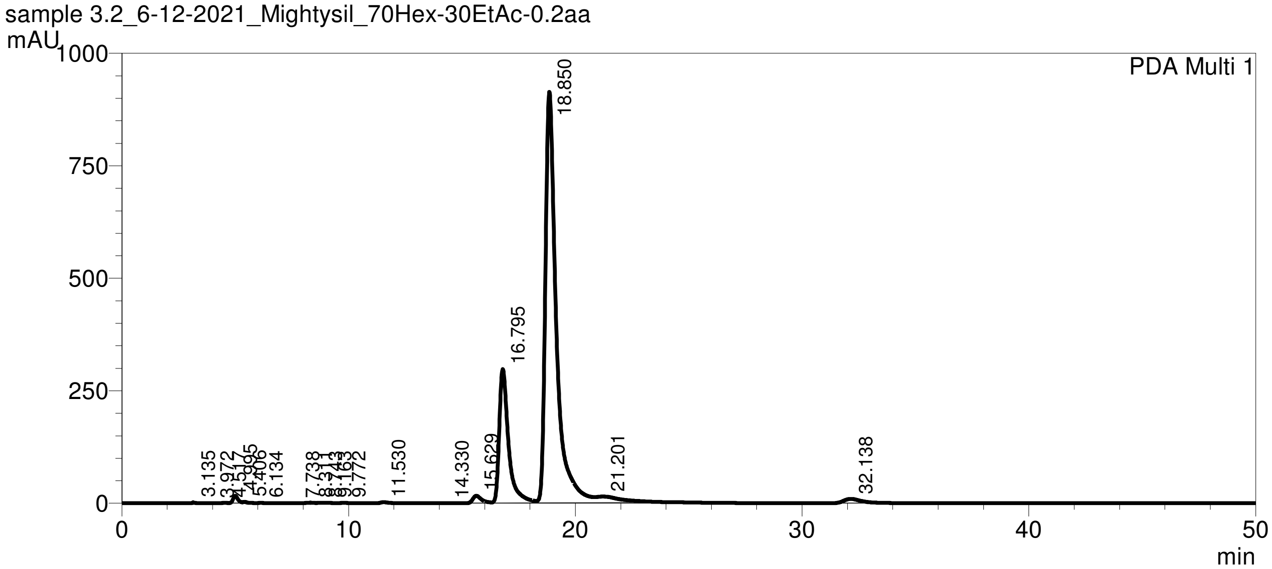

1 PDA Multi 1/320nm 4nm

PDA Ch1 320nm 4nm

Peak#	Ret. Time	Area	Height	Area %	Height %
1	3.135	12827	2370	0.031	0.187
2	3.972	7144	377	0.017	0.030
3	4.517	14188	985	0.034	0.078
4	4.995	248355	16367	0.596	1.289
5	5.406	40104	2864	0.096	0.226
6	6.134	8613	422	0.021	0.033
7	7.738	5472	375	0.013	0.030
8	8.311	22747	1142	0.055	0.090
9	8.743	20320	784	0.049	0.062
10	9.163	8231	528	0.020	0.042
11	9.772	15938	977	0.038	0.077
12	11.530	59275	2425	0.142	0.191
13	14.330	3859	229	0.009	0.018
14	15.629	446908	16249	1.072	1.280
15	16.795	8531579	297505	20.458	23.431
16	18.850	31567621	913506	75.696	71.945
17	21.201	146140	3004	0.350	0.237
18	32.138	543634	9615	1.304	0.757
Total		41702956	1269724	100.000	100.000
